# High-dimensional single-cell analysis of human natural killer cell heterogeneity

**DOI:** 10.1038/s41590-024-01883-0

**Published:** 2024-07-02

**Authors:** Lucas Rebuffet, Janine E. Melsen, Bertrand Escalière, Daniela Basurto-Lozada, Avinash Bhandoola, Niklas K. Björkström, Yenan T. Bryceson, Roberta Castriconi, Frank Cichocki, Marco Colonna, Daniel M. Davis, Andreas Diefenbach, Yi Ding, Muzlifah Haniffa, Amir Horowitz, Lewis L. Lanier, Karl-Johan Malmberg, Jeffrey S. Miller, Lorenzo Moretta, Emilie Narni-Mancinelli, Luke A. J. O’Neill, Chiara Romagnani, Dylan G. Ryan, Simona Sivori, Dan Sun, Constance Vagne, Eric Vivier

**Affiliations:** 1 Aix Marseille Université, CNRS, INSERM, Centre d’Immunologie de Marseille-Luminy, Marseille, France; 2 Leiden University Medical Center, Willem-Alexander Children’s Hospital, Laboratory for Pediatric Immunology, Leiden, the Netherlands; 3 Leiden University Medical Center, Department of Immunology, Leiden, the Netherlands; 4 Wellcome Sanger Institute, Wellcome Genome Campus, Cambridge, UK; 5 Biosciences Institute, Newcastle University, Newcastle upon Tyne, UK; 6 T Cell Biology and Development Unit, Laboratory of Genome Integrity, Center for Cancer Research, National Cancer Institute, National Institutes of Health, Bethesda, MD USA; 7 Center for Infectious Medicine, Department of Medicine Huddinge, Karolinska Institutet, Karolinska University Hospital, Stockholm, Sweden; 8 Department of Medicine Huddinge, Karolinska Institutet, Stockholm, Sweden; 9 Division of Clinical Immunology and Transfusion Medicine, Karolinska University Hospital, Stockholm, Sweden; 10 Sweden Broegelmann Research Laboratory, Department of Clinical Science, University of Bergen, Bergen, Norway; 11 Department of Experimental Medicine (DIMES), University of Genoa, Genoa, Italy; 12 Laboratory of Clinical and Experimental Immunology, IRCCS Istituto Giannina Gaslini, Genova, Italy; 13 Department of Medicine, University of Minnesota, Minneapolis, MN USA; 14 Department of Pathology and Immunology, Washington University School of Medicine, St. Louis, MO USA; 15 Department of Life Sciences, Imperial College London, Sir Alexander Fleming Building, South Kensington, London, UK; 16 Laboratory of Innate Immunity, Institute of Microbiology, Infectious Diseases and Immunology (I-MIDI), Campus Benjamin Franklin, Charité - Universitätsmedizin Berlin, Corporate Member of Freie Universität Berlin and Humboldt-Universität zu Berlin, Berlin, Germany; 17 Mucosal and Developmental Immunology, Deutsches Rheuma-Forschungszentrum (DRFZ), an Institute of the Leibniz Association, Berlin, Germany; 18 Department of Dermatology and NIHR Biomedical Research Centre, Newcastle Hospitals NHS Foundation Trust, Newcastle upon Tyne, UK; 19 Department of Immunology & Immunotherapy, The Marc and Jennifer Lipschultz Precision Immunology Institute, Icahn School of Medicine at Mount Sinai, New York, NY USA; 20 Department of Oncological Sciences, The Tisch Cancer Institute, Icahn School of Medicine at Mount Sinai, New York, NY USA; 21 Department of Microbiology and Immunology and the Parker Institute for Cancer Immunotherapy, University of California, San Francisco, San Francisco, CA USA; 22 Precision Immunotherapy Alliance, The University of Oslo, Oslo, Norway; 23 The Institute for Cancer Research, Oslo University Hospital, Oslo, Norway; 24 Tumor Immunology Unit, Bambino Gesù Children’s Hospital, IRCCS, Rome, Italy; 25 School of Biochemistry and Immunology, Trinity Biomedical Sciences Institute, Trinity College Dublin, Dublin, Ireland; 26 Institute of Medical Immunology, Charité Universitätsmedizin Berlin, Corporate Member of Freie Universität Berlin and Humboldt Universität zu Berlin, Berlin, Germany; 27 Innate Immunity, Deutsches Rheuma-Forschungszentrum Berlin (DRFZ), ein Leibniz Institut, Berlin, Germany; 28 Berlin University Alliance, Berlin, Germany; 29 MRC Mitochondrial Biology Unit, University of Cambridge, Cambridge, UK; 30 IRCCS Ospedale Policlinico San Martino, Genova, Italy; 31 Innate Pharma Research Laboratories, Innate Pharma, Marseille, France; 32 APHM, Hôpital de la Timone, Marseille-Immunopôle, Marseille, France; 33 Paris-Saclay Cancer Cluster, Le Kremlin-Bicêtre, France

**Keywords:** Innate lymphoid cells, RNA sequencing, Tumour immunology

## Abstract

Natural killer (NK) cells are innate lymphoid cells (ILCs) contributing to immune responses to microbes and tumors. Historically, their classification hinged on a limited array of surface protein markers. Here, we used single-cell RNA sequencing (scRNA-seq) and cellular indexing of transcriptomes and epitopes by sequencing (CITE-seq) to dissect the heterogeneity of NK cells. We identified three prominent NK cell subsets in healthy human blood: NK1, NK2 and NK3, further differentiated into six distinct subgroups. Our findings delineate the molecular characteristics, key transcription factors, biological functions, metabolic traits and cytokine responses of each subgroup. These data also suggest two separate ontogenetic origins for NK cells, leading to divergent transcriptional trajectories. Furthermore, we analyzed the distribution of NK cell subsets in the lung, tonsils and intraepithelial lymphocytes isolated from healthy individuals and in 22 tumor types. This standardized terminology aims at fostering clarity and consistency in future research, thereby improving cross-study comparisons.

## Abstract (web summary)

Single-cell technologies have unveiled a complex understanding of NK cells that has led to variations in nomenclature and inconsistencies across the scientific literature. Here, Vivier and colleagues used these technologies to dissect the heterogeneity of NK cells, revealing three prominent NK cell subsets in healthy human blood.

## Main

NK cells are lymphocytes of the innate immune system that belong to the ILC family^1^. NK cells were initially recognized for their capability to identify and eliminate virus-infected and tumor cells independently of prior sensitization, but their multifaceted roles have since been acknowledged. These include not only direct immune responses, but also regulatory functions that influence the adaptive immune system.

The heterogeneity of NK cells is central to their varied functions. Over time, researchers have identified distinct NK cell subgroups, each characterized by unique functional potentials and developmental pathways. These traditional classification methods mainly relied on surface marker expression. Along this line, human NK cells are typically divided into two main categories on the basis of the density of CD56, the 140-kDa isoform of the neural cell adhesion molecule (NCAM)^2^, on the cell surface: CD56^bright^ and CD56^dim^ NK cells. Further distinctions in the CD56^dim^ population are made on the basis of expression of the CD57 carbohydrate moiety^3^ on the cell surface and the absence of CD94–NKG2A and CD62L; cells with these features comprise a more mature subset^4–7^. Additionally, adaptive NK cells, which make up a distinct NK cell subset demonstrating characteristics akin to those of adaptive immune cells, emerge in certain immune contexts, such as human cytomegalovirus (HCMV) encounters^8,9^. The advent of advanced single-cell technologies, namely scRNA-seq and CITE-seq, has precipitated a paradigm shift in our understanding of NK cells. These technologies reveal that the NK cell landscape is more intricate and nuanced than previously understood and is marked by subtle distinctions. However, despite these advancements, a unified and standardized description of NK cell heterogeneity remains elusive. Current definitions vary between laboratories and could lead to discrepancies in scientific literature. This lack of standardized terminology creates major challenges, particularly in translating research across model systems or cohorts of people.

The increasing relevance of NK cells in therapeutic approaches, especially in NK-cell-based immunotherapy against cancer, underscores the necessity of a comprehensive understanding of their heterogeneity. Misinterpretation or neglect of specific NK cell subsets could have substantial implications, potentially affecting the effectiveness or safety of therapies. In this study, we integrated scRNA-seq and CITE-seq data from ~225,000 NK cells (718 donors) to establish a baseline classification of NK cells in the blood, lung, tonsil and intraepithelial lymphocytes of healthy individuals, and in 22 tumor types. These data were extracted from 7 distinct publicly available datasets. The accession code of each of the datasets used is listed in Supplementary Table 3 and ‘Data Availability’. This classification is intended to serve as a reference point for future studies, thereby facilitating a more standardized approach to understanding and using NK cells in both research and clinical settings.

## Results

### Human circulating NK cells comprise three main populations

**Fig. 1:**
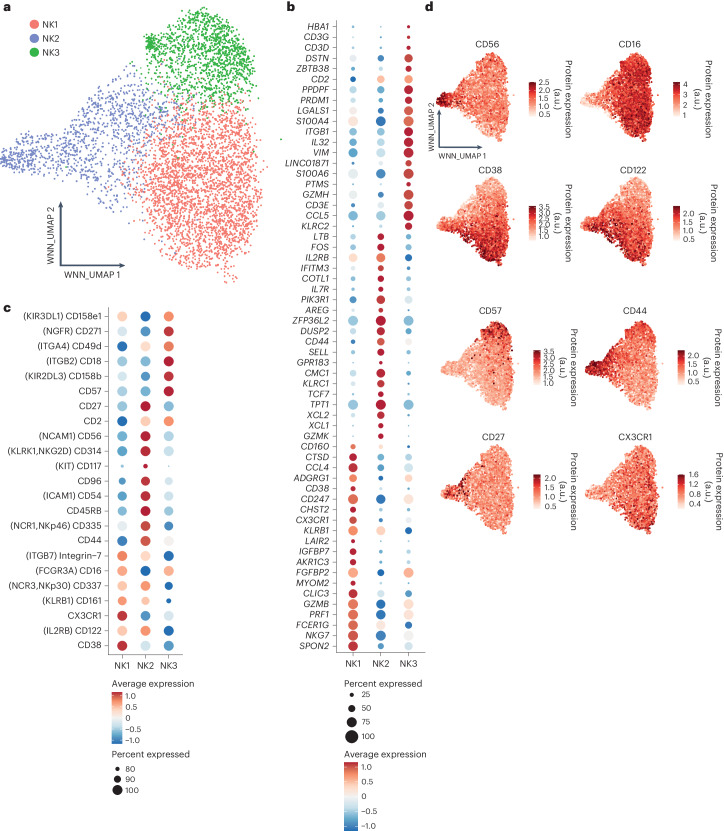
CITE-seq analysis reveals three prominent subsets of peripheral blood NK cells in healthy individuals. Based on dataset 5. **a**, WNN and UMAP (WNN_UMAP) visualization of NK cells sorted from healthy human blood with clusters identified by unsupervised hierarchical clustering (based on scRNA-seq and expression of 228 surface proteins). **b**, Dot plot of the 20 most distinguishing genes expressed for the three major subsets of human blood NK cells. Gene expression was analyzed using the using the two-sided Wilcoxon rank-sum test with Bonferroni adjustment. Ribosomal genes and mitochondrial genes were removed for clarity. The color indicates the *Z*-score scaled gene expression levels. **c**, Dot plot of the most distinguishing proteins expressed for the three major subsets of human blood NK cells. Protein expression was analyzed using the two-sided Wilcoxon rank-sum test with Bonferroni adjustment. Alternative protein names are shown in parentheses. The color indicates the *Z*-score scaled protein expression levels. **d**, WNN_UMAP visualization of the surface expression of the major discriminating proteins expressed at the surface of NK1, NK2 and NK3 cells. a.u., arbitrary units.

To systematically and comprehensively categorize human blood NK cells, we used a high-dimensional CITE-seq dataset, encompassing 228 antibody-derived tags (ADTs) and the transcriptional profiles of 5,708 NK cells from eight healthy donors^10^. To effectively integrate both RNA- and protein-expression data, we used the weighted nearest neighbors (WNN) method^10^. Initially, we isolated non-proliferating NK cells at the baseline and then reclustered them to elucidate the foundational heterogeneity among blood NK cells. Our analysis revealed three primary NK cell subsets: NK1, NK2 and NK3 (Fig. 1a). We subsequently analyzed their transcriptional (Fig. 1b) and proteomic signatures (Fig. 1c,d).

The NK1 cluster was marked by high protein expression of CD16, CX3CR1, CD161, β7-integrin and CD38 (Fig. 1c,d). Its transcriptional profile highlighted genes corresponding to these proteins, along with elevated levels of genes encoding cytotoxic molecules (*GZMB* and *PRF1*) and markers of NK cell maturity, such as *CD160*, *CD247*, *ADGRG1*, *NKG7*, *FCER1G*, *LAIR2*, *SPON2*, *CLIC3* and *CHST2* (Fig. 1b). Cells in the NK1 cluster express lower levels of CD56 compared to cells in the NK2 cluster and lower levels of CD57 compared to cells in the NK3 cluster.

The NK2 cluster was defined by high expression of CD56, CD27, CD44, CD54, CD45RB, CD314 (NKG2D) and CD335 (NKp46) and little or no expression of CD16 and CD57 at the protein level (Fig. 1c,d). At the transcriptome level, NK2 cells showed pronounced expression of ribosomal genes (RPL and RPS gene families, Supplementary Table 1) and genes encoding proteins involved in protein synthesis and structural integrity (*EEF1A1*, *TPT1*), indicative of heightened protein synthesis and proliferative capacity. This subset also expressed various genes encoding cytokine receptors (*IL2RB*, *IL7R*), membrane receptors (*KLRC1* encoding NKG2A), transcription factors (*TCF7*), soluble factors that modulate immune responses (*XCL1*, *XCL2*, *AREG*) and molecules implicated in cell migration and tissue homing (*CD44*, *GPR183*, *SELL*), along with granzyme K (*GZMK*) (Fig. 1b). Expression of the classic NK cell markers CD57 and CD16 was reduced or absent on NK2 cells compared with NK1 and NK3 cells, indicating that the NK2 population comprised CD56^bright^ and early-stage CD56^dim^ NK cells.

**Extended Data Fig. 1:**
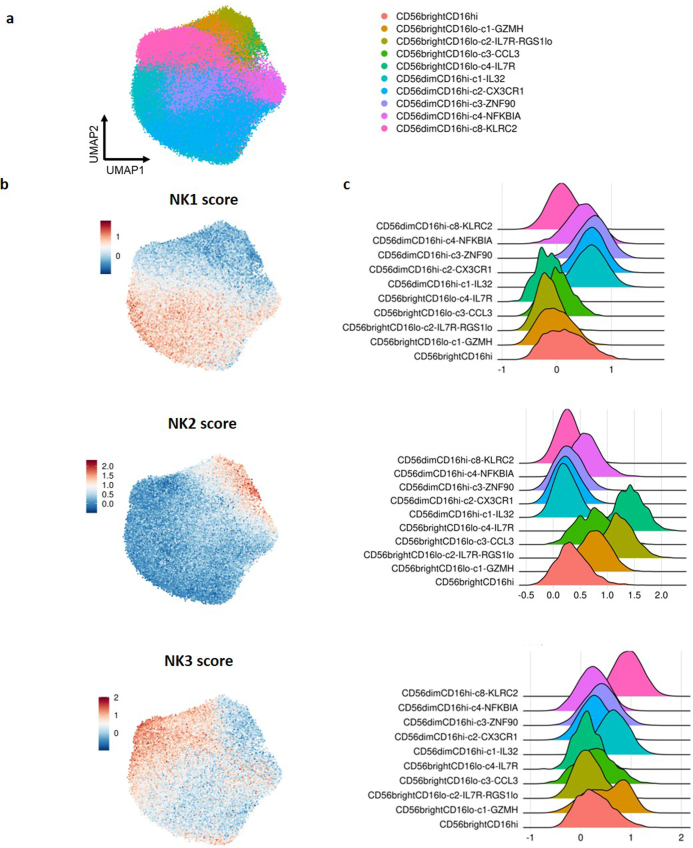
The classification of NK cells into 3 main families is robust in other blood NK cell atlas. Based on Dataset 6. **a**, Uniform Manifold Approximation and Projection (UMAP) of blood NK cells from Tang et al. pan-cancer NK cells atlas. Subsets constituting less than 1% of circulating NK cells were excluded which resulted in a total of 84,343 human blood NK cells for analysis. **b**, UMAP of blood NK cells from Dataset 5 scored with NK1, NK2, and NK3 signatures. **c**, RidgePlot visualization of the scoring of the clusters defined by Tang et al. (n= 676 samples).

**Extended Data Fig. 2:**
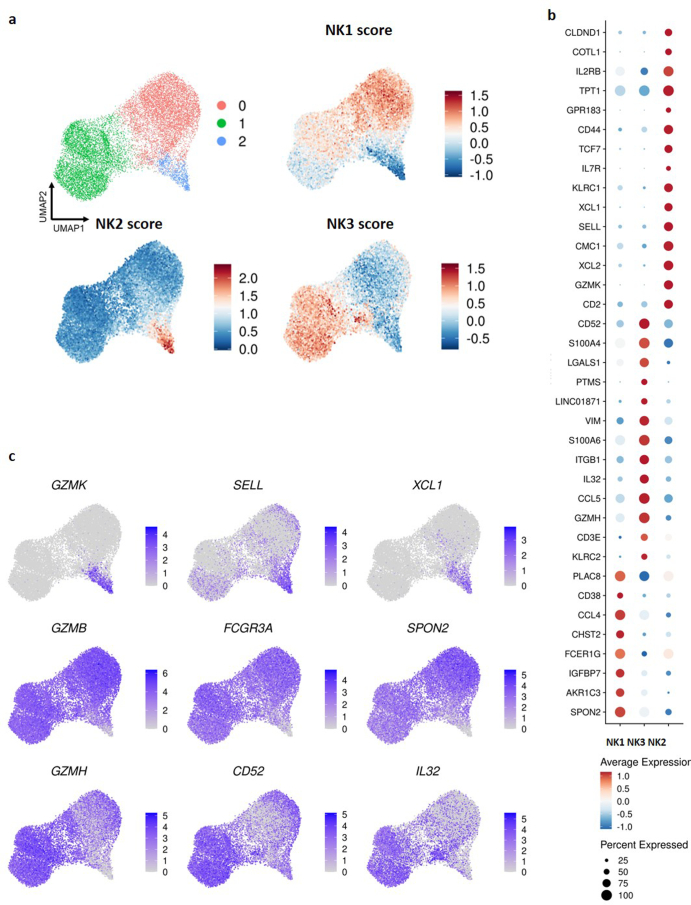
The classification of NK cells into 3 main families is robust in other blood NK cell samples. Based on Dataset 4b. **a**, UMAP based on 2 independent samples of NK cells sorted from healthy human blood with clusters identified by unsupervised hierarchical clustering and their scoring with NK1, NK2, and NK3 signatures. **b**, **c**, Dot plot and UMAP visualization of some of the most discriminatory markers expressed at the transcriptional level by the three major subsets of human blood NK cells.

For the NK3 cluster, the protein-expression profile included CD16, CD57, CD271 (*NGFR*), CD2, CD18, CD49d and inhibitory killer cell immunoglobulin-like receptors (KIRs) (CD158e, CD158b), with lower expression levels of CD56, NKp30, NKp46, CD161 and CD122 (Fig. 1c,d). Transcriptionally, NK3 cells were characterized by the preferential expression of genes encoding transcription factors (*PRDM1* (encoding BLIMP1) and *ZBTB38*), surface molecules and receptors (*CD2* and *KLRC2* (encoding NKG2C)), CD3 chain transcripts (*CD3D*, *CD3E*, *CD3G*), secreted cytokines and chemokines (*IL32*, *CCL5*) and granzyme H (*GZMH*) (Fig. 1b). Altogether, the combined protein and transcriptional signature of the NK3 cluster closely resembles that of adaptive NK cells, and this cluster’s preferential expression of CD57 and PRDM1 suggests that it also includes mature CD57^+^CD56^dim^ NK cells that are not produced in response to HCMV. We then confirmed the robustness of the classification of human blood NK cells into the NK1, NK2 and NK3 clusters by applying the derived transcriptional signatures to blood NK cells from other available datasets^11^ (Extended Data Figs. 1a–c and 2a–c).

### The three primary NK cell populations can be split into six subsets

**Fig. 2:**
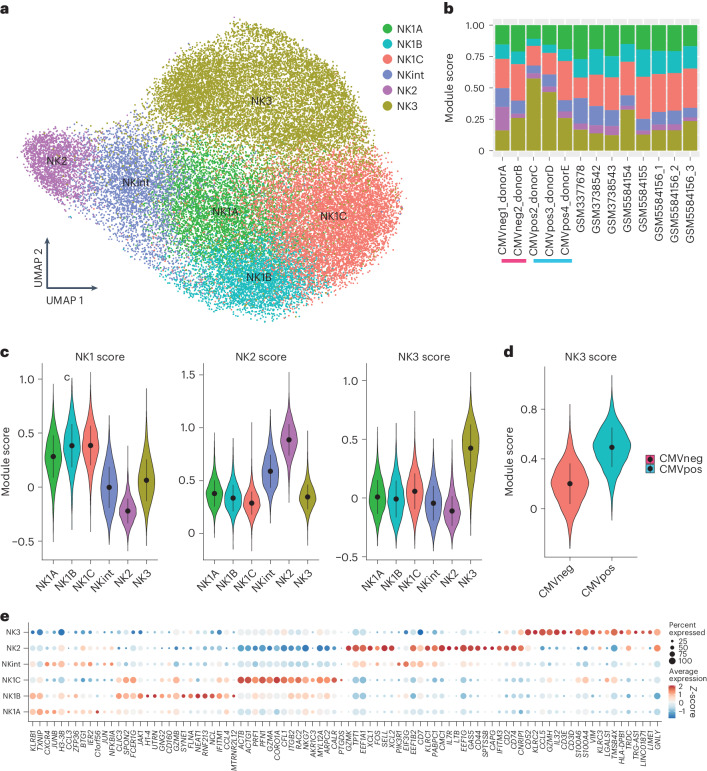
The three most important NK cell populations can be subdivided into six subgroups. Based on datasets 1–4a. **a**, UMAP visualization of NK cells sorted from healthy human blood, with clusters identified by unsupervised hierarchical clustering. **b**, Bar graph showing the proportion of cells within each cluster in all donors. Blue and pink bars are shown under HCMV-positive and HCMV-negative individuals, respectively. **c**, Violin plot of the scoring of the six NK clusters with respect to established NK1, NK2 and NK3 signatures (*n* = 13 samples). In the violin plots, the point is the median value. The error bars present the median +/- standard deviation. **d**, Violin plot of the scoring of the NK3 clusters with the NK3 signature in HCMV-positive and HCMV-negative individuals (*n* = 13 samples). **e**, Dot plot of the 20 most distinguishing genes for each subset of NK cells. Gene expression was analyzed using the two-sided Wilcoxon rank-sum test with Bonferroni adjustment. Ribosomal genes and mitochondrial genes were removed for clarity. The color indicates the *Z*-score scaled gene expression levels.

**Extended Data Fig. 3:**
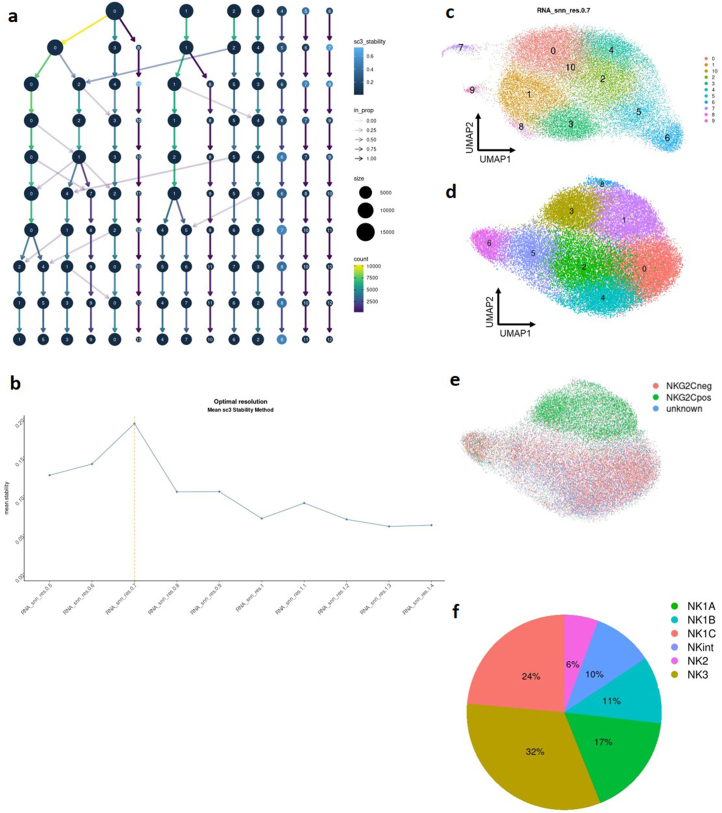
The NK cells in human blood can be divided into six subgroups. Based on Dataset 1,2,3 and 4a **a**, Clustree plot of sc3 stability of clusters at different clustering resolution (from k=0.5 to k=1.4). **b**, Mean sc3 stability as a function of the granularity of clustering resolution. **c**, UMAP visualization of the plot of NK cells sorted from healthy human blood with clusters identified by unsupervised hierarchical clustering at a granularity of 0.7 (optimal resolution according to sc3 stability). **d**, UMAP visualization of the subpopulations of NK cells from the blood of healthy individuals with clusters identified by unsupervised hierarchical clustering after removing proliferating cells and populations representing less than 3% of total NK cells. **e**, UMAP visualization of NKG2C protein expression. **f**, Pie chart showing the proportion of each subgroup in the NK cell population in blood.

To further delineate the heterogeneity of blood NK cells, we integrated scRNA-seq data from sorted NK cells from 13 healthy individuals across four datasets using the same RNA-seq protocol (10× genomics v2 chemistry protocol), therefore including 36,270 cells after high-quality cell filtering. This procedure resulted in the identification of eight well-defined clusters (Extended Data Fig. 3a–d). Three clusters (1, 3 and 8) shared an NK3 signature marked by genes such as *KLRC2* (encoding NKG2C), *CD52*, *IL32* and *GZMH* (Supplementary Table 1) and were enriched in cells expressing NKG2C on their surface (Extended Data Fig. 3e). Notably, NK3B (cluster 1) was distinguished by expression of members of the HLA-D gene family, *CD74*, *CCL5*, *CD7* and *KLRC1*, and NK3A exhibited enhanced cytotoxic capabilities (through expression of *GZMA*, *GZMB* and *PRF1*) (Supplementary Table 1). Previous data have shown that there is dramatic epigenetic and transcriptional heterogeneity within adaptive NK cells in HCMV^+^ individuals. This heterogeneity was observed within the same person and across different people, reflecting the clonality of adaptive NK cells rather than functionally distinct programs^12^. We consolidated these three clusters into a single cluster for subsequent analyzes. This led to a final configuration of six clusters (Fig. 2a and Extended Data Fig. 3f). The integrated dataset in our study can be explored at: https://collections.cellatlas.io/meta-nk.

**Extended Data Fig. 4:**
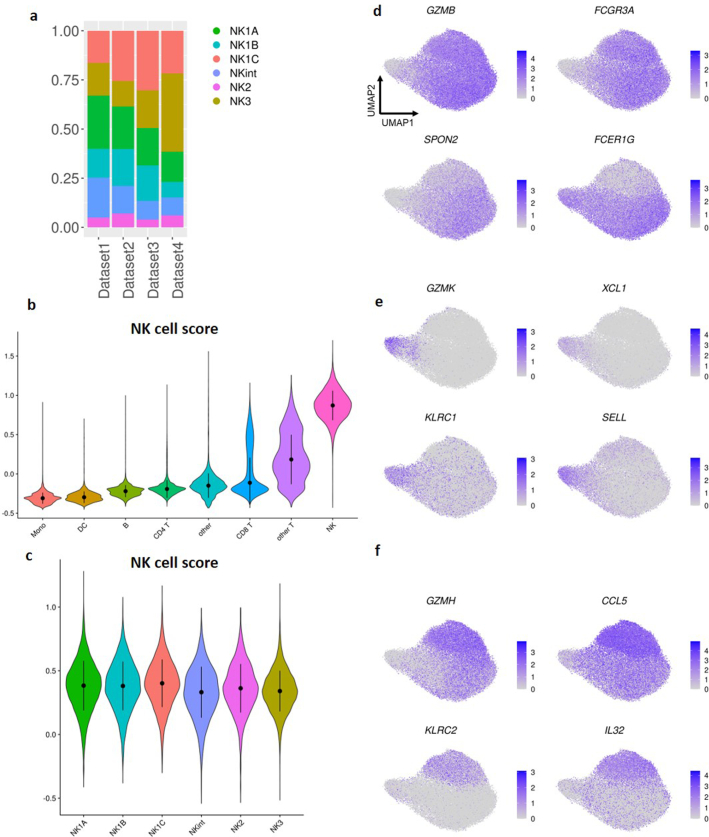
The NK cells in human blood can be divided into six subgroups. a,c and d-f: Based on Based on Dataset 1,2,3 and 4a b: Based on Dataset 5. **a**, Bar graph showing the proportion of cells within each cluster in the datasets. (n= 13 samples) **b**, Violin plot of the scoring of all CD45^pos^ cells from Dataset 5 with the 13 genes characteristic of human NK cells as defined by Crinier et al. The error bars present the median +/- standard deviation. (n= 8 samples) **c**, Violin plot of the scoring with the 13 genes characteristic of human NK cells as defined by Crinier et al. The error bars present the median +/- standard deviation. (n= 13 samples) **d, e, f**, UMAP visualization of the expression of some key markers of NK1, NK2 and NK3 populations.

Upon confirming that batch correction was adequate and ensuring that our final cluster designations were free from batch effects both at the dataset (Extended Data Fig. 4a) and donor (Fig. 2b) levels, we scored all CD45^pos^ populations from dataset 5 with the previously defined^13^ 13-gene signature (*CD160*, *CD244*, *CHST12*, *CST7*, *GNLY*, *IL18RAP*, *IL2RB*, *KLRC1*, *KLRC3*, *KLRD1*, *KLRF1*, *PRF1*, *XCL2*) that is characteristic of human NK cells, therefore validating the ability of this signature to discriminate NK cells from other subsets (Extended Data Fig. 4b). We also used the 13-gene signature to score the six subsets of NK cells, thus verifying the robustness of this signature across all NK populations (Extended Data Fig. 4c). Then, we evaluated each cluster against established transcriptional signatures for NK1, NK2 and NK3 (Fig. 2c). Three clusters (cluster 2, cluster 4 and cluster 0) exhibited a strong correlation with the NK1 signature, prompting their reclassification as NK1A, NK1B and NK1C, respectively. As expected, the cluster containing the NK3 subpopulations displayed a clear association with the NK3 signature. Notably, a higher NK3 score was observed in NK3 cells derived from HCMV^+^ individuals (Fig. 2d). Simultaneously, cluster 6 showed a strong correlation with NK2 signature, justifying its classification as NK2. Finally, cluster 5 displayed an intermediate association with both NK1 and NK2 signatures and was thus renamed intermediate NK (NKint). This reassignment is consistent with the gene-expression patterns delineated by pre-defined signatures, facilitating a clearer understanding of the functional landscape within the blood NK cell repertoire.

Analysis of the top 20 defining markers for the six clusters (Fig. 2e) provided a detailed transcriptional profile for each cluster. NK1 cells, as noted in Figure 1b, showed a core signature indicative of chemokines (*CCL3*, *CCL4*) and proteins critical for cytotoxicity and its regulation (*PRF1*, *GZMA*, *GZMB*, *NKG7*), cytoskeletal dynamics (*RAC2*, *ARPC2*, *CFL1*) and cellular adhesion (*ITGB2*, *CALR*). NK1 subpopulations expressed unique subset-specific markers. NK1A was characterized by high expression of *CXCR4* and the *JUN* and *JUNB*, which encode AP-1 transcription factors; NK1B was distinguished by the surface marker *CD160*, the long non-coding RNA *NEAT1* and the interferon-induced transmembrane protein 1 *IFITM1*; NK1C exhibited enhanced cytotoxic potential, with higher levels of granzyme and perforin transcripts, a distinct expression profile related to prostaglandin metabolism (*PTGDS*, *AKR1C3*) and the most active cytoskeletal profile (*ACTB*, *ACTG1*, *CFL1*, *RAC2*, *ARPC2*).

NK2 and NKint populations, whose core signatures shared genes encoding chemokines (*XCL1*, *XCL2*), granzymes (*GZMK*), proteins involved in transcription and signaling regulation (*NFKBIA*, *FOS*, *BTG1*, *GAS5*) and protein synthesis (*TPT1*, *EEF* gene family) and surface proteins (*CD44*, *CD74*, *CD7*, *KLRC1*), also displayed distinct markers. NK2 expressed *LTB*, *SELL*, *GNLY* and *IL7R*, whereas NKint exhibited strong expression of *CXCR4*, *JUNB*, *ZFP36*, *IER2* and *EIF3G*.

NK3, along with the previously defined signature (*KLRC2*, *CCL5*, *GZMH*, *IL32*, *CD3E*, *CD3D*, *S100A4*, *LGALS1*), expressed additional markers (*CD52*, *TMSB4X*) and shared certain ones with the NK2 population, such as NKG2E (*KLRC3*) and granulysin (*GNLY*). This intricate transcriptional landscape underscores the diverse functionalities and regulatory mechanisms at play within the NK cell subsets.

Further investigation into the distribution of these populations among the 13 healthy donors revealed a predominance of NK1 cells, constituting approximately 60% ± 12% of circulating NK cells. NK2 and NK3 cells represented 17% ± 7% and 24% ± 14%, respectively (Supplementary Table 2). A more granular analysis at the subpopulation level (Fig. 2b and Extended Data Fig. 3f) showed that nearly half of the NK1 population was made up of NK1C cells, translating to 26% ± 6% of all circulating NK cells. The NK2 population represented a minor fraction of total NK cells (6% ± 4%) as compared to NKint (11% ± 4% of total NK cells). The NK3 cluster, characterized by distinctive expression of markers indicative of both adaptive and terminally mature NK cells (such as *PRDM1* and *B3GAT1* (encoding an enzyme key for the biosynthesis of CD57)) along with genes uniquely associated with adaptive NK cells (*CD3E* and *ZBTB38*) (Fig. 1c,d), exhibited considerable variability in its prevalence across individuals (Fig. 2b). Our study’s approach to cluster identification was conducted without consideration of HCMV status. Consequently, to discern the potential impact of HCMV on the NK3 cluster, we conducted separate analyzes of the frequency and predictive scores of NK3 cells in individuals positive for HCMV (HCMV^+^) and in those without HCMV (HCMV^−^). Notably, cells in the NK3 cluster were observed in both HCMV^+^ and HCMV^−^ donors (Fig. 2b and Extended Data Fig. 3f). However, a higher NK3 score was predominantly observed in NK3 cells derived from HCMV^+^ individuals (Fig. 2d). Altogether, these insights provide a quantitative perspective on the distribution and variability of NK cell subsets in the bloodstream.

### Molecular features of NK cell subsets

**Fig. 3:**
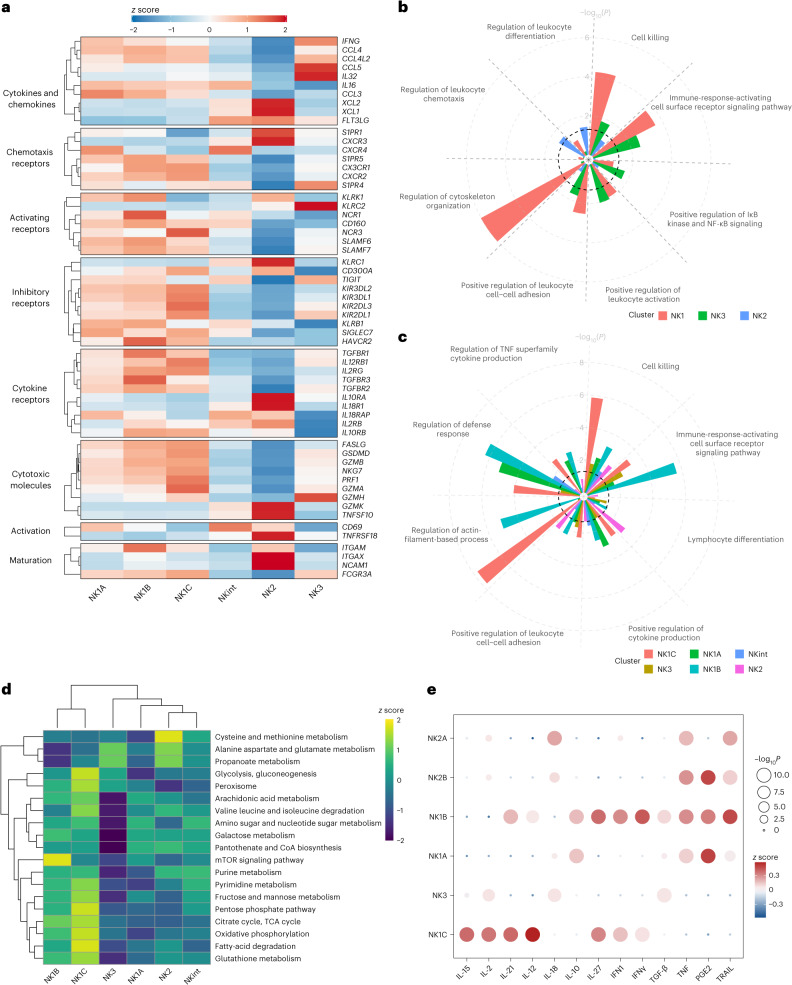
Markers of interest, functions and metabolism characterizing NK cell populations. Based on datasets 1–4a. **a**, Heatmap showing the differential expression of markers of interest among NK cell subsets. The color scale is based on *z*-score-scaled gene expression. The z-score distribution ranges from −2 (blue) to 2 (red). **b**,**c**, Selected GO terms showing enrichment in the three major populations (and six major subsets) of healthy human blood NK cells. Benjamini–Hochberg-corrected −log_10_(*P*) values were calculated by a hypergeometric test. The black dotted line indicates the significance threshold, which is −log_10_(0.05). **d**, Heatmap showing the differential enrichment for selected metabolic pathways among NK cell subsets. The color scale is based on the *z* score of the normalized enrichment score for each metabolic pathway. **e**, Assessment (*z* scores) of the response to different cytokines and chemokines in each subgroup, quantified by Cytosig. IFN, interferon; PGE2, prostaglandin E2. *P* values were computed by comparing *z* scores in one NK subset with those in other subsets using two-sided Student’s *t*-tests, and –log_10_(*P*) values exceeding 10 were capped at 10 to facilitate visualization.

After confirming that the six subpopulations expressed the populations’ defining markers (Extended Data Fig. 4d–f), we computed the *z* scores for the expression levels of various pertinent markers in the NK cell subpopulations, considering only those genes expressed above a defined threshold—detection in more than 5% of circulating NK cells—for inclusion in the heatmap. This approach yielded a heatmap that, beyond previously identified markers, unveiled additional distinctive characteristics for the subpopulations (Fig. 3a).

We examined cytokine and chemokine production and found that NK1 subpopulations were characterized by robust transcription of *CCL4*, *CCL3*, *CCL4L2* and *IL16*, whereas NK2 and NKint cells exhibited predominant transcription of *FLT3LG* along with *XCL1* and *XCL2*. Finally, NK3 cells were marked by high transcription levels of *IFNG*, *IL32* and *CCL5*. Differential expression was also apparent in chemotaxis receptors and cell–cell adhesion proteins. In particular, the subsets were distinguished by different patterns of sphingosine-1-phosphate receptors (*S1PR1* for NK2, *S1PR4* for NK3 and *S1PR5* for NK1). Furthermore, the CXC chemokine receptor family had a role in distinguishing the subpopulations (*CXCR2* and *CX3CR1* for NK1, *CXCR3* for NK2 and *CXCR4* for both NK1A and NKint). Classic activating receptors of NK cells also exhibited subset-specific expression patterns. High levels of NKp46 (*NCR1*), *CD160*, NKp30 (*NCR3*) and signaling lymphocyte activation molecule receptor genes were characteristic of the NK1 population. NK2 shared a pronounced expression of NKG2D (*KLRK1*) with NK1A and NK1B. As expected, NKG2C (*KLRC2*) was predominantly expressed by the NK3 population. Inhibitory-receptor expression profiles diverged between subsets. NK1 cells expressed higher levels of inhibitory KIRs, along with TIM3 (*HAVCR2*), CD161 (*KLRB1*) and *SIGLEC7*, whereas NK2 and NKint cells had an elevated expression level of NKG2A (*KLRC1*), and NK3 cells appeared have higher expression levels of *TIGIT*. In terms of cytokine-receptor expression, NK1 populations exhibited heightened expression levels of *TGFBR1*, *TGFBR2* and *TGFBR3*, as well as *IL12RB1*, *IL10RB* and *IL2RG*. By contrast, NK2 cells were characterized by a preference for *IL2RB* (consistent with their strong expression of CD122 at the protein level, Fig. 1c), *IL10RA*, and a distinct expression of *IL18R* and its accessory protein *IL18RAP*. These findings suggest that the subsets have varying levels of sensitivity to cytokines and chemokines.

The observed cytotoxic profiles were in line with prior observations: NK1 populations exhibited a spectrum of cytotoxic molecules and associated proteins—*GZMA*, *GZMB*, *PRF1*, *NKG7*, *GSDMD* and *FASLG*—whereas NK2 and NKint displayed strong expression of *GZMK* and *TRAIL* (*TNFSF10*). The NK3 subset expressed intermediate levels of cytotoxic molecules and was distinguished by high *GZMH* expression.

Activation markers also served as differential markers, with *CD69* being most prominent in NK1B and NKint, whereas *TNFRSF18* (encoding GITR) was more pronounced in NK2. Moreover, classic markers of NK maturation aligned with earlier descriptions: CD56 (*NCAM1*) was more prevalent in NK2, and CD16 (*FCGR3A*) expression increased progressively from NK2 to NK1C. In addition, CD11B (*ITGAM*) levels were higher in NK1B, whereas CD11C (*ITGAX*) expression was more pronounced in NK2.

Gene Ontology (GO) term enrichment analysis revealed distinct functional specializations within NK subpopulations. NK1 cells were primarily involved in processes such as cell–cell adhesion, activation response, signaling, cytoskeletal activity and cell-mediated cytotoxicity (Fig. 3b). These findings underscore NK1 cells’ have pivotal cytotoxic effector functions. By contrast, NK2 cells were linked to enhanced chemotaxis regulation and leukocyte differentiation, suggestive of their ability to infiltrate tissues and an ongoing maturation process. NK3 cells displayed an upregulation in leukocyte activation. We then explored the functions of the six main NK cell subpopulations (Fig. 3c). NK1B cells were found to be highly responsive to activation through surface receptors, indicating their potential as primary targets in immunotherapeutic strategies. Both NK1A and NK1B populations were significantly enriched for production of tumor necrosis factor (TNF) and cytokines. Notably, the NK1C subset seems to be the most cytotoxic, as indicated by its pronounced cytoskeletal activity and cell-killing signature. An intriguing discovery was the considerable enrichment of tricarboxylic acid (TCA) cycle activities in the NK1C subset, prompting further investigation using single-cell gene set variation analysis (scGSVA).

**Extended Data Fig. 5:**
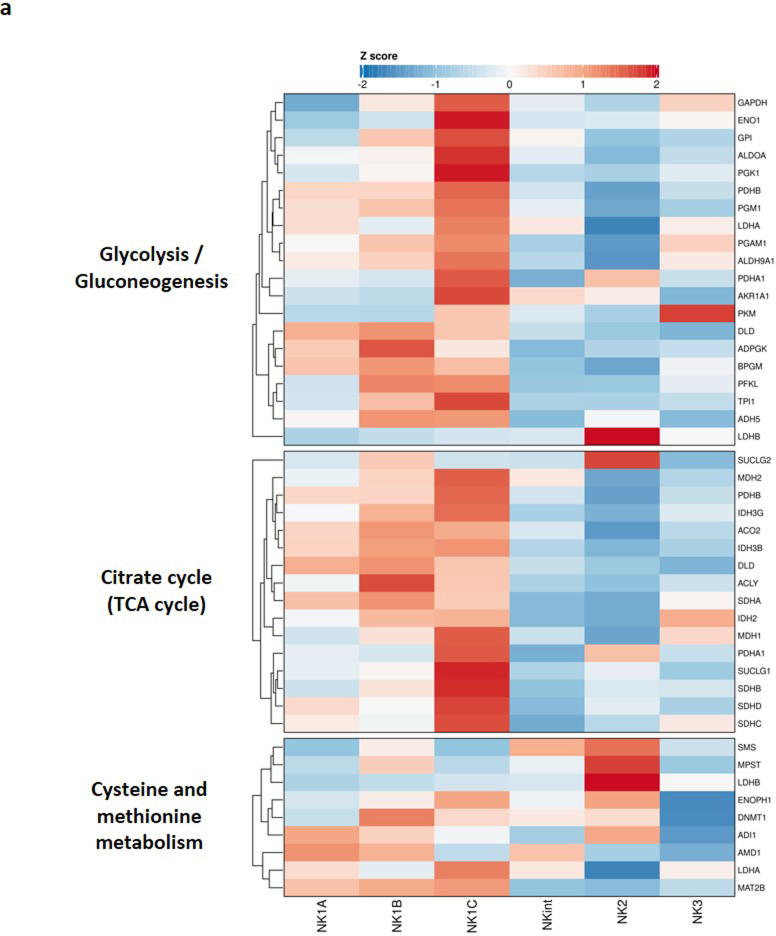
Markers of interest, functions and metabolism characterizing NK cell populations. Based on Dataset 1,2,3 and 4a. **a**, Heatmap showing the differential expression of the genes composing three metabolic pathways of interest among NK cell subsets. The color scale is based on z-score-scaled gene expression. The z-score distribution ranges from −2 (blue) to 2 (red).

Clustering analysis based on metabolic-pathway-enrichment analysis separated the NK cell subpopulations into two broad categories, with NK1B and NK1C clustering more closely together and separately from the NK1A, NK2, NK3 and NKint subsets (Fig. 3d). The NK1C subset appears to be ‘hypermetabolic,’ with notable enrichment across the central carbon metabolism, including glycolysis and the TCA cycle, and mitochondrial oxidative phosphorylation (OXPHOS), which could support enhanced cytotoxic activity. Similarly, the NK1B subset also exhibits enrichment in the TCA cycle and OXPHOS, albeit to a lesser extent than does NK1C, in contrast to the other NK cell subpopulations. In addition, NK1B cells are more clearly defined by an enrichment in the mTOR signaling pathway. Finally, cysteine and methionine metabolism are enriched in the NK2 subset. Decomposition analysis of this pathway (Extended Data Fig. 5a) found that the signature was in part driven by high expression levels of lactate dehydrogenase B (LDHB), spermine synthase (SMS) and 3-mercaptopyruvate sulfurtransferase (MPST). LDHB (which preferentially converts lactate to pyruvate and NAD^+^ to NADH) seems to be the predominant isoform of lactate dehydrogenase in the NK2 subset; LDHA (which preferentially converts pyruvate to lactate and NADH to NAD^+^) is highly expressed across the other NK subpopulations. These data suggest that different metabolic profiles underlie NK cell subpopulations and warrant further investigation.

To elucidate the varying responses of the six NK cell subsets to cytokine stimulation, we used the cytokine signaling analyzer (CytoSig)^14^, which predicts the responsiveness of cells to cytokine signals. This analysis indicated that the NK2 population exhibits a pronounced reaction to interleukin-18 (IL-18), consistent with the strong expression of *IL18R* and its associated protein *IL18RAP* in this subset (Fig. 3e). NKint, NK1A and NK1B cells seemed to be more susceptible to IL-10 and PGE2, which are signals that can dampen immune responses, in particular in the tumor microenvironment^15,16^. By contrast, NK1B and NK3 showed a greater response to transforming growth factor beta (TGF-β). TGF-β is notorious for its immunosuppressive effects on NK cells^17^, particularly within the tumor microenvironment, where it can hinder their cytotoxic functions^18^. Consistent with previous studies, NK3 showed reduced sensitivity to IL-12 (ref. ^9^). Finally, NK1C cells demonstrated the most robust response to a suite of cytokines, namely IL-2, IL-15 and IL-12, that is traditionally associated with the activation and proliferation of NK cells^15^.

### Transcriptional trajectories of NK cell subpopulations

The comprehensive examination of six NK cell subsets has revealed not only their distinctive characteristics in terms of markers, cytokine response and functionalities, but also a continuum in their transcriptional landscapes, particularly between the NKint and NK1A subsets. This continuum seems to bridge the transcriptional states of NKint with NK1C. To investigate the potential transcriptional pathways connecting these subsets, we performed a multifaceted analysis.

**Fig. 4:**
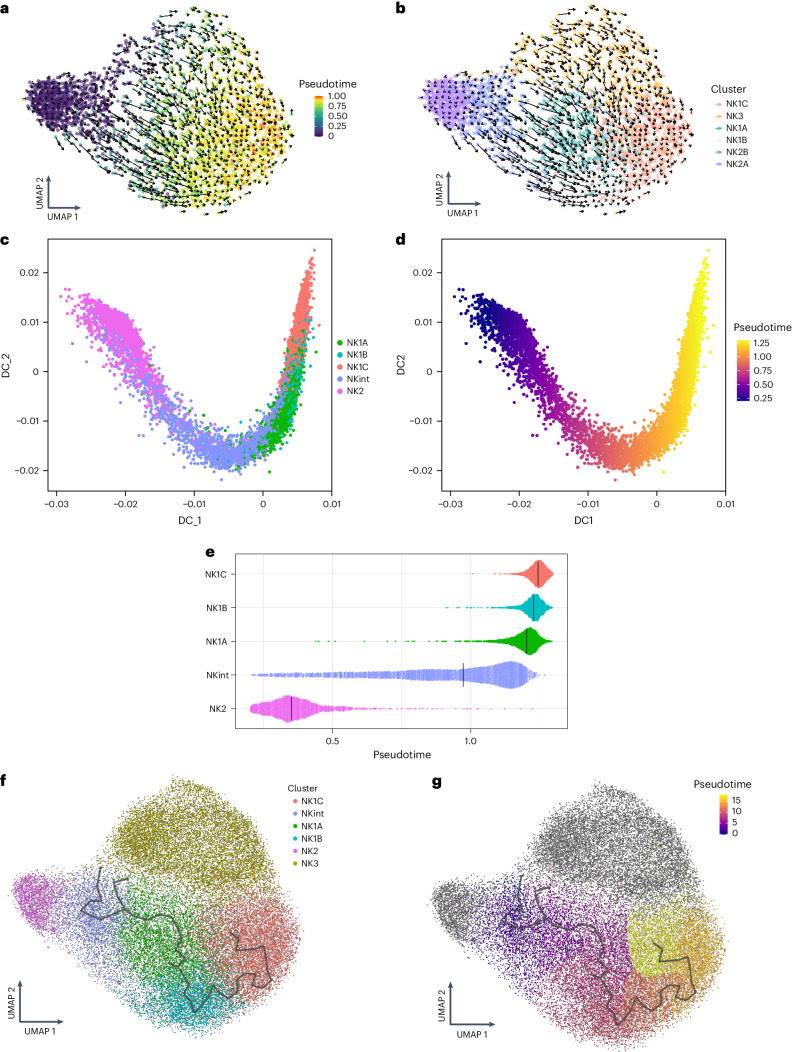
Putative transcriptional trajectories connecting NK cell subpopulations. **a**–**e**, Based on dataset 4a. **f**–**g**, Based on datasets 1–4a. **a**,**b**, RNA-velocity analysis and pseudotime inference based on velocity analysis in a representative sample. **c**,**d**, Confirmation of trajectories and pseudotime analysis using a diffusion-map approach (Destiny analysis of dataset 4a). **e**, Plot of pseudotime derived from diffusion-map analysis across all NK cell subgroups. **f**, Monocle-derived trajectory performed on the NK1A, NK1B and NK1C subsets and projected onto the UMAP, colored by clusters. **g**, Monocle-derived trajectory performed on NK1 subpopulations and projected onto the UMAP colored by pseudotime (inferred by Monocle3).

First, RNA velocity was used to predict the future states of individual cells. This analysis indicated that the majority of NK2 cells would likely persist as NK2 cells, forming a specific NK2 trajectory (Fig. 4a,b). However, it also pointed towards a potential differentiation pathway from NKint into NK1C. This path was characterized by a clear pseudotime progression from NKint to NK1C, transitioning through intermediary populations (NK1A and NK1B) (Fig. 4a,b). NK3 cells, which exhibit clonal-like transcriptional dynamics owing to their interaction with HCMV^12^, were excluded from the following trajectory analysis, to avoid having their unique transcriptional behavior skew the findings. Further trajectory analysis using diffusion maps (Destiny^19^) and trajectory inference (Monocle3 (ref. ^20^)) corroborated the pathway suggested by the RNA-velocity analysis (Fig. 4c–g). Pseudotime inference clearly outlined a trajectory from NKint to NK1C (Fig. 4d,e,g). Notably, the pseudotime inferred through diffusion-map analysis highlighted a considerable gap between NK2 and NKint (Fig. 4e), reinforcing the concept of two distinct trajectories: one in which NK2 cells predominantly remain NK2, and another leading from NKint to NK1C. This latter trajectory aligns with the metabolism-based unsupervised clustering previously discussed (Fig. 3d), which grouped NK1B and NK1C closely together owing to their strong central carbon metabolism activity. By contrast, NKint and NK1A clustered together and exhibited lower metabolic activity.

**Extended Data Fig. 6:**
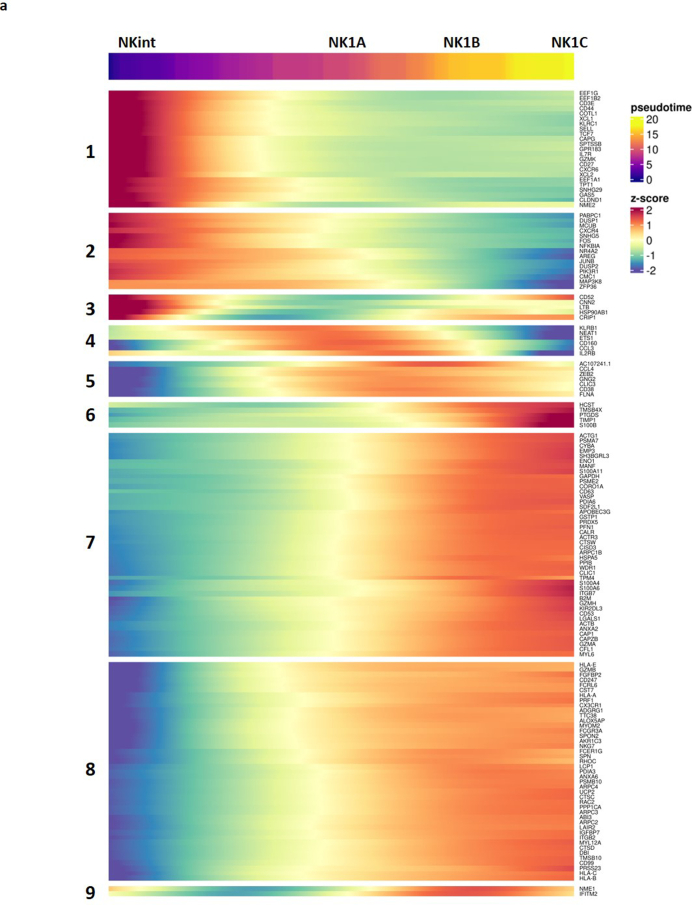
Dissection of the trajectory leading from NKint to NK1C. Based on Dataset 1,2,3 and 4a. **a**, Dynamic heatmap of the evolution of the top 150 markers that evolve most along the pseudotime of the trajectory leading from the NKint subset to the NK1C subset.

Building on the Monocle analysis, we homed in on the top 150 genes that exhibited significant changes along the NK cell maturation trajectory from NKint to NK1C, as indicated by a *q* value below 0.05 and a high Moran’s *I* correlation score. This detailed examination revealed nine gene modules, each of which was sequentially activated as the cells progressed through maturation stages (Extended Data Fig. 6a).

**Fig. 5:**
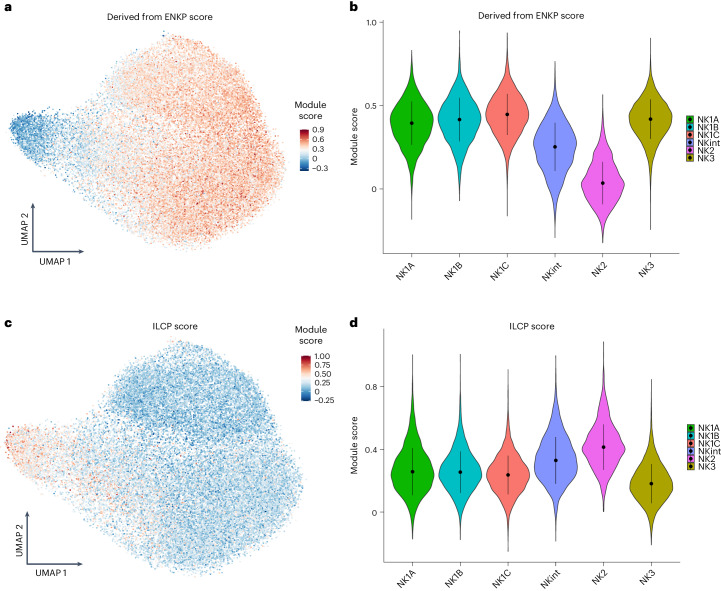
Putative ontogeny of the main NK populations. Based on datasets 1–4a. **a**, UMAP visualization of the module score of individual cells scored with signatures derived from the main NK cell progenitor (ENKP) identified in mice. **b**, Violin plots of the module scores of individual cells scored with ENKP signatures. Data are shown as median ± s.d. (*n* = 13 samples). **b**,**d**, In the violin plots the point is the median value. The error bars present the median +/- standard deviation. **c**, UMAP visualization of the module score of individual cells scored with human blood ILCP signatures. **d**, Violin plots of the module scores of individual cells scored with signatures of human blood ILCPs Data are shown as median ± s.d. (*n* = 13 samples).

The RNA-velocity analysis also predicts another major developmental pathway, indicating that a considerable portion of NK2 cells is likely to maintain the NK2-cell state. Consistent with this possibility, evidence has been presented suggesting that mouse NK cell populations arise from two distinct lineages: a primary progenitor, known as the early NK cell progenitor (ENKP), and an alternative one, called the innate lymphoid common progenitor (ILCP), which is also capable of giving rise to other types of ILCs^21^. By mapping the transcriptional module scores of human blood ENKPs onto a uniform manifold approximation and projection (UMAP) representation, we observed that both NK1 and NK3 populations displayed transcriptional signatures that closely align with those of NK cells originating from ENKPs (Fig. 5a,b). In addition, the scoring of NK1, NK2 and NK3 subsets on the basis of recently available blood human ILCP signatures^22^ revealed that their signature is enriched in NK2s (Fig. 5c,d). Altogether, these observations support the existence of two divergent ontogenic pathways: one for NK1 and NK3, originating from ENKPs, and another for NK2, originating from ILCPs.

**Extended Data Fig. 7:**
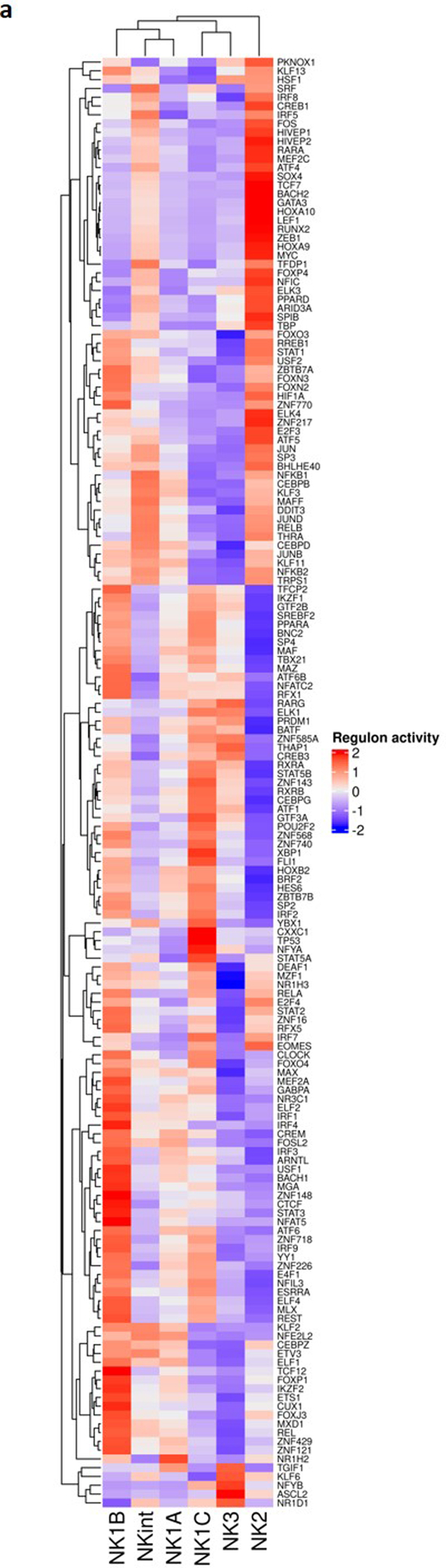
Master regulators genes characteristic for each subset of NK cells in the blood and putative ontogeny of the main NK populations. Based on Dataset 1,2,3 and 4a. **a**, Heatmap showing the differential expression of true transcription factors detected in NK cell subsets. The color scale is based on the z-score of the regulon activity. The z-score distribution ranges from −2 (blue) to 2 (red).

To elucidate the master regulatory genes that define the six NK cell subpopulations, we conducted a gene regulatory network analysis using the single-cell regulatory network inference and clustering (SCENIC) workflow^23^. The initial step involved cataloging the regulons identified in our dataset. Each regulon consists of a transcription factor or cofactor and its associated target genes (Extended Data Fig. 7a). Next, we compared our list of regulons with a more robust database of verified transcription factors^24^. This comparison was crucial for excluding unreliable transcription factors and proteins that bind to RNA and DNA non-specifically, and to focus our analysis solely on bona fide transcription factors. Unsupervised clustering based on regulon activity first revealed two striking features: first, that NK2 branched away from the other subsets, supporting the theory of a distinct ontological origin for NK2. Second, clustering first grouped the NKint and NK1A subpopulations, suggesting that they might represent early stages of NK1 cell differentiation, then grouped the NK1B cells that appeared more differentiated, and finally included the NK1C and NK3 subsets that correspond to more advanced NK cell states.

This sequential differentiation pattern highlights the complex regulatory mechanisms that govern NK cell development and differentiation. Transcription factors that are pivotal in NK cell maturation^25^, such as T-bet (*TBX21*) and BLIMP1 (*PRDM1*)^26,27^, showed a progressive increase across NK2, NKint, NK1A, NK1B to NK1C continuum. Conversely, *MYC*, *TCF7*, *RUNX2* and *GATA3* were predominantly expressed in NK2 subsets, aligning with previous research findings^28^. NK3 was distinguished by robust expression of *ASCL2* and *KLF6*, and the continued presence of BLIMP1 (*PRDM1*). Therefore, the observed expression pattern of key master regulators of maturation substantiates the hypothesis that there are distinct lineages of NK cell progenitors.

### Distribution of NK cell subsets in healthy tissue

**Fig. 6:**
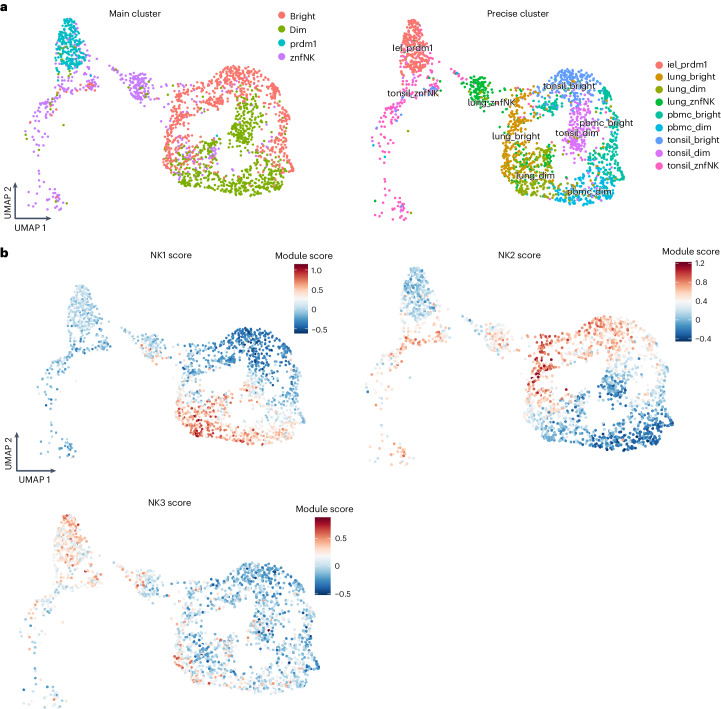
Distribution of NK1, NK2 and NK3 cell subsets in tissues. Based on dataset 7. **a**, UMAP visualization of the main populations of group 1 ILCs present in PBMCs, tonsil, lung and IEL, colored by their main populations and by their cluster and tissues (as defined in dataset 7). **b**, UMAP visualization of the module score of individual cells scored with signatures of NK1, NK2 and NK3 of ILC populations present in tonsil, lung and IELs. iel_prdm1, Intestinal intraepithelial lymphocytes; PRDM1+ NK cells; lung_bright, lung CD56bright NK cells; lung_dim, lung CD56bright NK cells; lung_znf NK, lung ZNF683+ NK cells; pbmc_bright, blood CD56bright NK cells; pbmc_dim, blood CD56dim NK cells; tonsil_bright, tonsil CD56bright NK cells; tonsil_dim, tonsil CD56dim NK cells; tonsil_znfNK, tonsil ZNF683+ NK cells.

**Extended Data Fig. 8:**
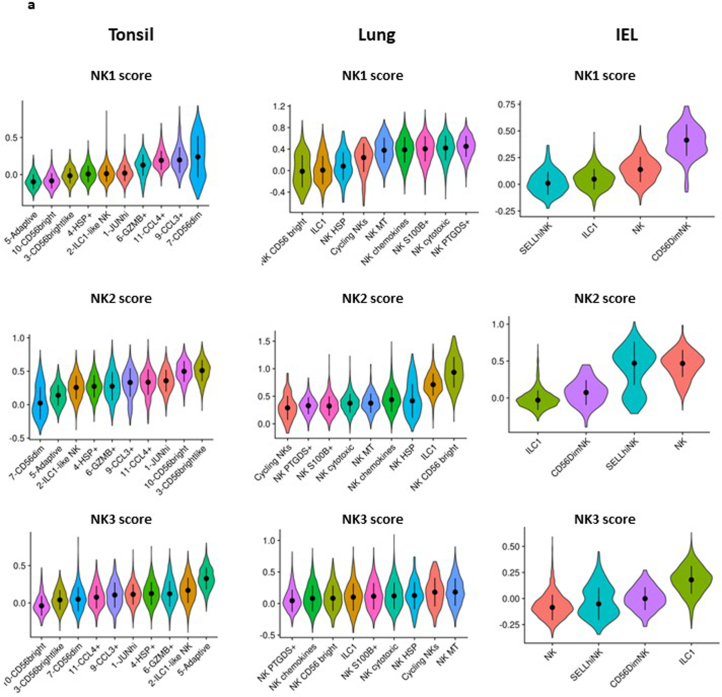
Distribution of NK1, NK2 and NK3 cell subsets in tissues. Based on Dataset 7. **a**, ViolinPlot visualization of the module score of individual cells scored with signatures of NK1, NK2 and NK3 of ILC populations present in tonsil, lung and IELs and grouped by clusters (as defined in Dataset 7). The error bars present the median +/- standard deviation. (Lung: n = 4 samples, Tonsil: n = 6 samples, IEL: n = 4 samples).

NK cells are found in tissues in addition to the peripheral blood^29^. The link between circulating NK cells, tissue-infiltrating NK cells and tissue-resident ILCs is an emerging area of research. ILCs vary greatly depending on their environment and the local signals, such as cytokines, that they are exposed to, resulting in distinct ILC profiles in different tissues and diseases^1^. A detailed description of these ILC variations was recently published^22^. We therefore analyzed the scRNA-seq data in the earlier study^22^ to investigate the distribution of NK1, NK2 and NK3 subsets in tonsils, lungs and intraepithelial lymphocytes (IELs) isolated from healthy individuals (Fig. 6a). Remarkably, the NK1 and NK2 signatures coincided with the CD56^dim^ and CD56^bright^ subsets, respectively, identified in lung, tonsil and IELs (Fig. 6b). More specifically, the vast majority of the different subgroups of CD56^dim^ and CD56^bright^ cells in these tissues could be characterized as NK1 and NK2, respectively (Extended Data Fig. 8a). A few discrete subsets of NK cells in the tonsils (labeled JUNhi, ILC1-like NK, HSP^+^) and lungs (labeled cyclic NKs, NK HSP, ILC1) could not be assigned to the NK1, NK2 or NK3 subsets. Because we had removed two subsets, cyclic NK cells and NK cells that exhibited characteristics of stress, for the analysis that led to the identification of the NK1, NK2 and NK3 subsets, we expected that some subsets, namely tonsil JUNhi, tonsil HSP^+^, lung NK HSP and cyclic NKs in the lung, could not be annotated. Notably, the tonsil ILC1-like and lung ILC1 subsets also did not match any of the NK1, NK2 and NK3 profiles, confirming that the later transcriptomic signatures preferentially resemble those of NK cells. However, the partial enrichment of lung ILC1s with NK2 signatures reinforces the idea that there is a shared ontology between these two populations (Extended Data Fig. 8a). Finally, our data show the similarities between the IEL ILC1 and NK3 subsets (Fig. 6a,b and Extended Data Fig. 8a), as illustrated in particular by the strong expression of PRDM1 in both the IEL ILC1 and NK3 subsets. These results indicate that the similarities between IEL ILC1s and NK cells and the divergence of IEL ILC1s from other tissue-resident ILC1s should be reanalyzed.

### Distribution of NK cell subsets in cancer

**Fig. 7:**
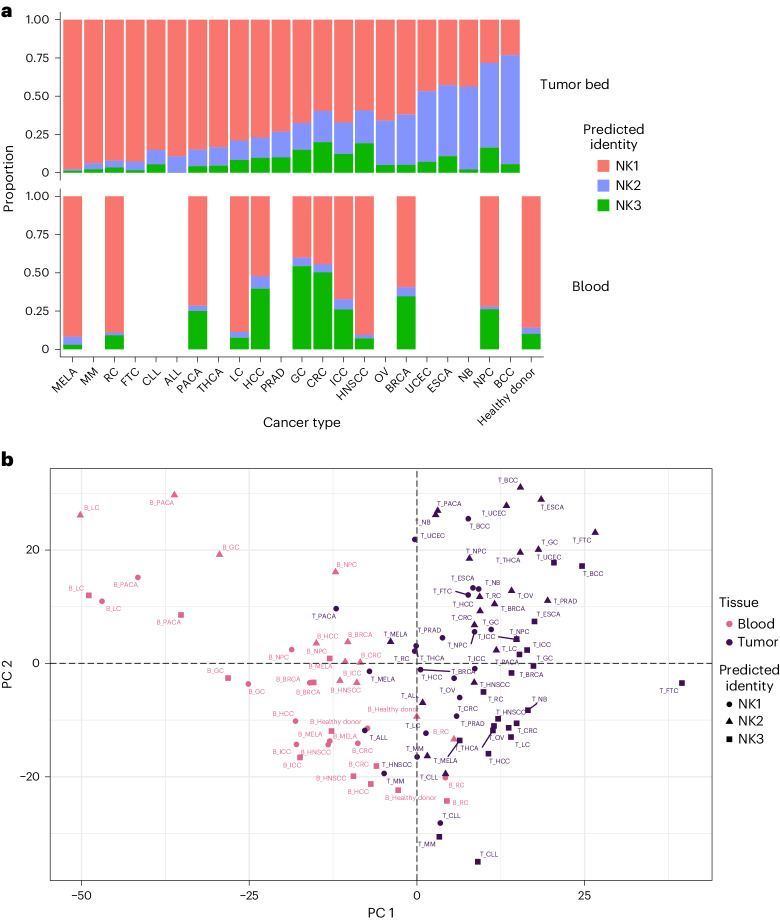
Distribution of NK1, NK2 and NK3 cell subsets in the blood of people with cancer and at the tumor bed. Based on datasets 1–4a and 6. **a**, Bar graph showing the proportion of the three main NK populations in the blood and at the tumor bed in 22 cancer types (*n* = 676 samples). **b**, PCA on tumor-infiltrating and blood NK cells, grouped by NK population, cancer conditions and tissue. The PCA is based on the mean expression levels of the 2,000 genes most differentially expressed across tissue and conditions. Groups are colored on the basis of their tissue of origin. PC1 and PC2 explained 13.7% and 11% of the variance, respectively (*n* = 676 samples). MELA, melanoma; MM, multiple myeloma; RC, renal carcinoma; FTC, fallopian tube carcinoma; CLL, chronic lymphocytic leukemia; ALL, acute lymphocytic leukemia; PACA, pancreatic carcinoma; THCA,: thyroid carcinoma; LC, lung cancer; HCC, hepatocellular carcinoma; PRAD, prostate cancer; GC, gastric cancer; CRC, colorectal cancer; ICC, intrahepatic cholangiocarcinoma; HNSCC, head and neck squamous cell carcinoma; OV, ovarian cancer; BRCA, breast cancer; UCEC, uterine corpus endometrial carcinoma; ESCA, esophageal cancer; NB, neuroblastoma; NPC, nasopharyngeal carcinoma; BCC, basal cell carcinoma.

**Fig. 8:**
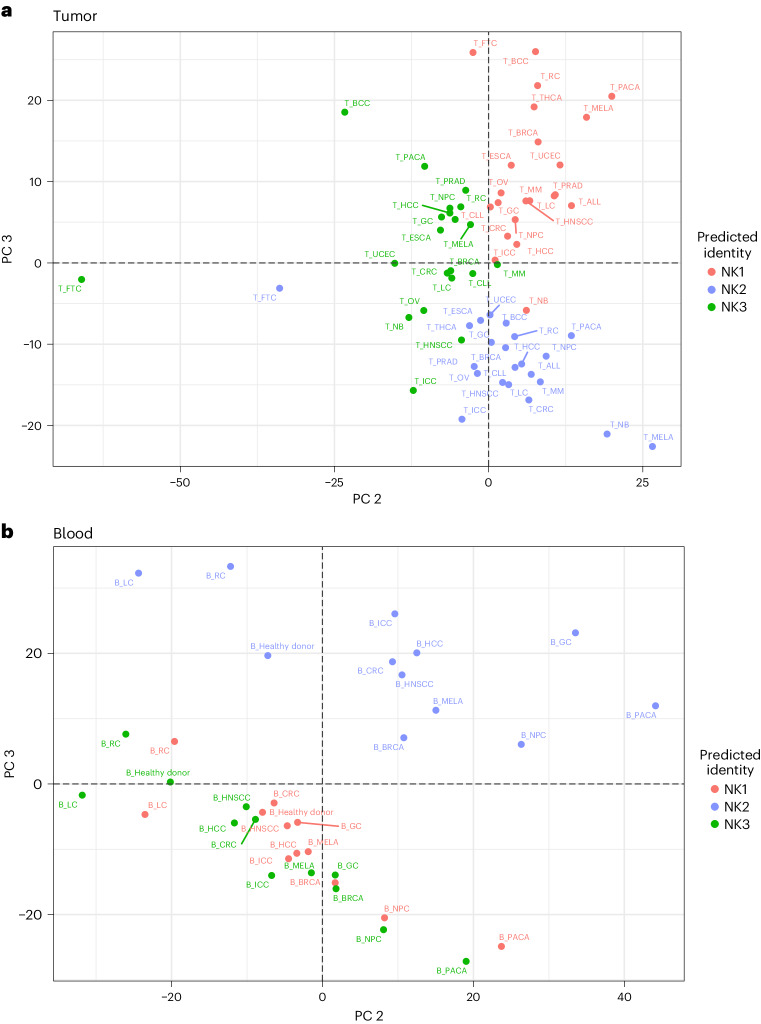
Distinct transcriptional phenotypes of NK1, NK2 and NK3 cell subsets in the blood of people with cancer and at the tumor bed. Based on datasets 1–4a and 6. **a**, PCA of blood NK cells, grouped by NK population and cancer conditions. The PCA is based on the mean expression levels of the 2,000 genes most differentially expressed across tissue and conditions. Groups are colored on the basis of NK cell subsets. PC2 and PC3 explained 8.1% and 7% of the variance, respectively (*n* = 676 samples). **b**, PCA of tumor-infiltrating NK cells, grouped by NK population and cancer conditions. The PCA is based on the mean expression levels of the 2,000 genes most differentially expressed across tissues and conditions. Groups are colored on the basis of NK cell subsets. PC2 and PC3 explained 13.9% and 12.7% of the variance, respectively (*n* = 676 samples).

**Extended Data Fig. 9:**
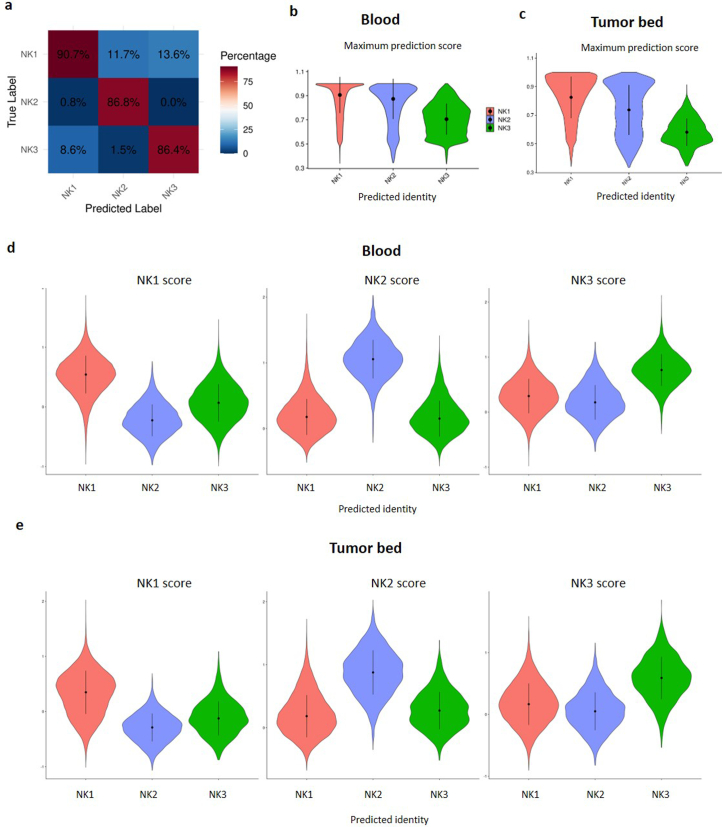
Distribution of NK1, NK2 and NK3 cell subsets in the blood of cancer patients and at the tumor bed. a: Based on dataset 1,2,3,4a. b-e: Based on dataset 6. **a**, Heatmap depicting accuracy of the label transfer for subset annotation tested on 20 % of the cells heldout to train the reference. **b**, ViolinPlot visualization of the maximum prediction score per cell in the blood NK samples of Dataset 6. Cells are grouped by their predicted identity. The error bars present the median +/- standard deviation. **c**, ViolinPlot visualization of the maximum prediction score per cell in the tumor-infiltrated NK samples of Dataset 6. Cells are grouped by their predicted identity. The error bars present the median +/- standard deviation. **d**, ViolinPlot visualization of the module score of individual blood NK cells of Dataset 6 scored with signatures of NK1, NK2 and NK3. Cells are grouped by their predicted identity. The error bars present the median +/- standard deviation. **e**, ViolinPlot visualization of the module score of individual tumor-infiltrated NK cells of Dataset 6 scored with signatures of NK1, NK2 and NK3. Cells are grouped by their predicted identity. The error bars present the median +/- standard deviation.

**Extended Data Fig. 10:**
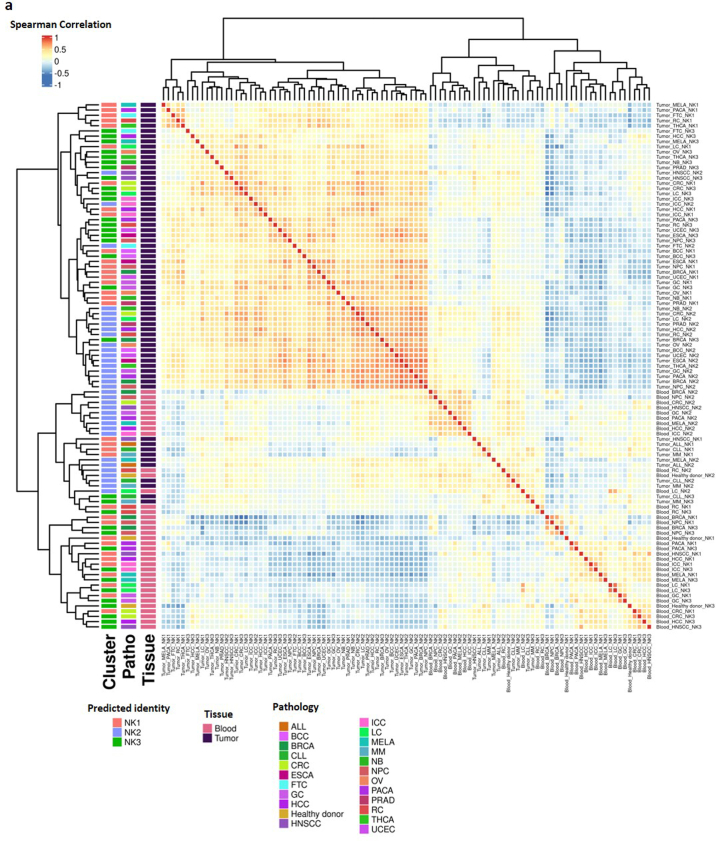
Distinct transcriptionnal phenotypes of NK1, NK2 and NK3 cell subsets in the blood of cancer patients and at the tumor bed. Based on dataset 6. **a**, Heatmap showing the Spearman correlation between NK1, NK2 and NK3 populations in healthy individuals and across 22 different cancer types in both blood and tumor. The error bars present the median +/- standard deviation. (n= 676 samples).

An important point of our analysis was to provide a benchmark for future comparisons with diseased conditions. Therefore, we analyzed the distribution patterns of NK1, NK2 and NK3 cell subsets in 22 cancer types (Fig. 7). To that end, we used a classical label-transfer approach (see Methods). After verifying the accuracy of the method used to annotate the subgroups (Extended Data Fig. 9a–e), we investigated the proportions and transcriptional proximity of these subsets across tissues and cancer types. The distribution of NK cell subsets in these 22 tumors varies by tumor type (Fig. 7a, top panel). This distribution does not correlate with that found in the blood (Fig. 7a, bottom panel). This difference between circulating and tumor-associated NK cells was confirmed by principal component analysis (PCA) (Fig. 7b, PC1), as was the accuracy of NK1, NK2 and NK3 annotation at the tumor bed (Fig. 8a,b, PC2 and PC3). The divergence between NK2 and the other subsets was also confirmed in blood from people with cancer, but the influence of the tumor on the distinction between the NK1, NK2 and NK3 subsets is stronger at the tumor bed than in the blood (Fig. 8a,b). The Spearman correlation calculated across NK groups, type of cancer and tissues and their unsupervised hierarchical clustering confirmed that NK cells first segregate by tissue type and then by the subset to which they belong (Extended Data Fig. 10a). The better grouping of NK1s, NK2s and NK3s in the tumor bed than in the blood also suggests an exacerbated phenotype in tumor conditions.

## Discussion

Although scRNA-seq and CITE-seq have considerably advanced exploration of the diversity of human NK cells, definitions of their cell types and subtypes have varied across publications for several reasons, including differing experimental protocols, data-acquisition methods and analysis tools. This has led to complexity in the literature and even disagreement as to whether certain cell subsets are real or artifacts arising from a particular processing methodology. For example, what seems to be an NK cell subtype could be the result of a stress response that was triggered during cell isolation or by culture conditions. Thus, it is important to establish a consensus framework for a basic set of NK cell types by pooling datasets obtained from multiple laboratories and analyzing them holistically.

The integration of CITE-seq and scRNA-seq NK cell data in our meta-analysis, including data from a total of more than 225,000 NK cells, led us to discern three major NK cell populations in peripheral blood, herein called NK1, NK2 and NK3. These populations are highly enriched in canonical CD56^dim^, canonical CD56^bright^ and HCMV-driven adaptive NK cells, respectively. The gene-expression profile of the NK1 population described here aligns with that of the previously described hNK_Bl1 cells, with strong expression of *FGFBP2*, *GZMB*, *SPON2* and *FCGR3A*^13^. The gene-expression profile of the NK2 population overlaps with that of hNK_Bl2, defined by high levels of *COTL1*, *CD44*, *XCL1*, *LTB* and *GZMK*. Notably, the equivalents of NK1 and NK2 have also been characterized in mouse blood: mNK_Bl1 and mNK_Bl2, respectively^13^. NK3 cells exhibited a pattern of gene expression overlapping with that of previously described HCMV-driven adaptive NK cells, defined by high levels of *KLRC2*, *CD3E* and *ZBTB38* (refs. ^12,30^). However, although these adaptive genes are the main drivers of the NK3 cluster signature, cells assigned to the NK3 cluster in our study can be also found at lower frequencies in HCMV^–^ individuals. Therefore, the NK3 cluster defined here is not limited to HCMV-driven adaptive NK cells. Additionally, considering that the transcriptional signature that is exclusive to adaptive NK cells is limited relative to the level of epigenetic remodeling that these cells undergo, the next step in resolving adaptive NK cell identity might be at the epigenetic level through single-cell ATAC sequencing methods^12^. This highlights the benefits of combining multimodal single-cell approaches when defining distinct cell subsets. Alternatively, adaptive NK cells can also be distinguished from canonical CD56^dim^ NK cells on the basis of deficient PLZF expression^8,9^.

The NK1 population could be reliably divided into three subsets, called NK1A, NK1B and NK1C; an NKint population with an intermediate phenotype between NK1 and NK2 was also characterized. In line with our meta-study, recently published scRNA-seq datasets also delineated multiple subclusters. The NK1A subset exhibited the highest expression levels of *CXCR4*, *JUN* and *JUNB*, mirroring the description of the previously published active CD56^dim^ (ref. ^31^) and intermediate CD56^dim^ (ref. ^32^) clusters. *CD160* and *IFITM1* were most abundant in the NK1B subset, a population that was predicted to have the highest response to chemokines and cytokines. The NK1C subset displayed the highest expression levels of *PRF1*, *PFN1*, *ACTB* and *NKG7*, concordant with the descriptions of mature and terminal CD56^dim^ (ref. ^31^), late CD56^dim^ (ref. ^32^), cluster 2 (ref. ^33^) and CD56^dim^CD57^+^ NK cells^5,30,34^.

Previous studies have reported an intermediate subset linking CD56^bright^ (NK2) and CD56^dim^ NK cells^12,31,32,35^. This intermediate subset shares a core signature including expression of *CD44*, *XCL1* and *GZMK*, but is distinguished by elevated expression of *CXCR4*, in line with our description of NKint. The high expression of KLRC1 (NKG2A) but low expression of CD56 indicates that the NKint population has strong similarities with the early NKG2A^+^KIR^−^CD56^dim^ NK cell population^5,30^. The intermediate expression of CD56, which lies between that of NK2 and NK1 and the lower levels of perforin and granzyme B, as well as the expression of CD27, which was detected at the beginning of the putative transcription pathway connecting NKint to NK1C, also point to a previously defined CD27^+^CD56^dim/bright^CD94^+^ NK population^36^.

Our comprehensive trajectory studies revealed two distinct developmental pathways for NK cells. The first trajectory indicates a path through which NK2 cells can maintain their identity; the second involves a progressive maturation process in which NKint cells evolve into the NK1A, NK1B and NK1C stages. For NK2 cells, which seem to derive from ILCPs, it is noteworthy previous research has identified a medullary population of human NK progenitors, termed NK0 (ref. ^37^), and that the human NK0 signature matches that of ILCPs, suggesting that NK0s might correspond to medullary ILCPs. As with NK1 cells, their maturation is associated with a notable shift in the transcriptional landscape, characterized in particular by an increase in expression of cytotoxicity-related genes such as *GZMA*, *GZMB* and *PRF1*. Concurrently, we observed an escalation in central carbon metabolism activities along this maturation trajectory. This escalation is marked by enhanced glycolysis, TCA cycle activity and OXPHOS. The module score analysis further corroborates these distinct developmental paths, revealing a strong association between the NK2 population and blood ILCPs, as evidenced by their shared signature markers including *SELL*, *CD44*, *LTB*, *IL7R* and *GPR183* (Figs. 2e and 5c,d). Similarly, NK1 and NK3 populations show a pronounced connection to ENKPs, aligning with the observation that Ly49H^+^ NK cells in mice, which respond to mouse CMV and are analogous to the human adaptive NK cells included in the NK3 subset, predominantly originate from ENKPs^21^. At the level of transcription factors, NK1 and NK3 have unique characteristics akin to certain ENKP traits, such as reduced expression of *GATA3*, *EOMES* and *TCF7* alongside an increased expression of *KLF2*. This multifaceted analysis underscores the intricate pathways and mechanisms governing NK cell differentiation and functionality.

Our investigation also sheds light on several molecular dimensions of NK cell biology, warranting additional research. A key finding is the distinct profiles of granzymes and perforin across the three primary NK populations. NK1 cells exhibit robust expression of *GZMA*, *GZMB* and *PRF1*, which have been extensively studied^38,39^. Conversely, NK2 cells predominantly express *GZMK*, known for its role in caspase-independent apoptosis^40,41^ and in controlling autoimmunity^42^. The NK3 subset is characterized by expression of *GZMH*, encoding granzyme H, which also initiates caspase-independent cell death^43^ and is effective in inducing rapid apoptosis in tumor cells^44^. This underlines the considerable antitumor potential of NK3 cells. But more remains to be understood about the biology of granzymes, as illustrated by the recent demonstration of the role of granzyme A in triggering production of gasdermin-B^45^. Another noteworthy finding was the cytokine profile of the NK2 subset that predominantly transcribed *FLT3LG* along with *XCL1* and *XCL2*, which encode proteins that attract dendritic cells and promote their antigen-presentation function^46,47^. The integrin profile of the NK1 subset and the change in their expression along the NK1-maturation trajectory suggest that these integrins could be instrumental in enhancing contact interactions with other cells, regulating NK1 cytotoxicity or facilitating NK1 cells’ entry into tissues (for example, ITGB7 dimerizes with ITGA4 to adhere to MAdCAM-1 for intestinal entry)^48^. A better understanding of the mechanism of expression and regulation of these integrins could have major clinical applications, such as enhancing antitumor immunity in colorectal cancer^49^.

Our results also indicate that there are notable differences in cytokine responses among NK cell subsets. In the context of adoptive NK cell therapy, IL-21 conditioning enhances proliferation, cytotoxicity and production of interferon-γ and TNF in NK cells^50^. The stronger response of NK1B and NK1C subsets to IL-21 makes them particularly promising for NK cell-based therapies. Additionally, IL-15 has been shown to boost NK cell metabolism and longevity^51^, aligning with the characteristics of the NK1C subset, which exhibits strong metabolic activity and a pronounced response to IL-15. These cytokine responses can be further exploited through the use of cytokine-armed NK cell engagers^52^, enhancing our understanding of subset-specific responses to improve and diversify these new therapeutic approaches.

Our data also show that the gene profiles of the NK1, NK2 and NK3 subsets extend beyond the peripheral blood of healthy individuals, and allowed us to describe the heterogeneity of NK cells in tissues. Indeed, we were able to identify NK1, NK2 and NK3 cell subsets in the lung, tonsils and IELs. The relevance of NK1, NK2 and NK3 profiles was also illustrated by distinguishing between subsets of tissue-infiltrating NK cells and ILC1s.

Notably, we were also able to analyze the distribution of NK cell subsets in 22 cancer types. This showed that the distribution of NK cell subsets varies depending on the tumor type and does not show a strict correlation with the distribution in the blood. The immediate implication of this observation is the relative value of monitoring NK cells in peripheral blood to assess NK cell immunity in people with cancer. Interestingly, the proportion of NK2 cells was increased in most tumors tested, particularly in ovarian cancer, breast cancer, endometrial carcinoma of the uterus, esophageal cancer, neuroblastoma, nasopharyngeal carcinoma and basal cell carcinoma. Although NK cell dysfunction at the tumor bed is well established^53^, no specific profile corresponding to dysfunctional NK cells has been characterized. The reported impairment of the cytolytic capacity of NK cells at the tumor bed is consistent with a shift towards the NK2 profile, in which the expression of molecules involved in the cytolytic machinery is low. It is also important to consider the metabolic profile of NK2 and its response to cytokines compared with NK1 and NK3. In particular, several therapeutic agents have been developed to stimulate NK cells using cytokines or mutant cytokines^54^, such as NK cell engagers armed with IL-2 variants^52^, and it is crucial to consider the cytokine sensitivity of tumor-associated NK cells.

The NK cell atlas presented here not only serves as a reference for future studies on NK cells in blood in health and disease, but is also a tool for understanding NK cell diversity in tissues in relation to circulating NK cells, the ontogeny of NK cells in tissues and the relationship between NK cells and ILC1s in tissues in health and disease.

## Methods

### scRNA-seq data retrieval and preprocessing

For datasets 1–4, scRNA-seq data were retrieved from the studies referenced in Supplementary Table 3. Single-cell sequencing data were aligned with the GRCh38 human reference genome and quantified using Cell Ranger (v6.1.2, 10x Genomics). The preliminary filtered data generated from Cell Ranger were used for downstream filtering and analyzes. First, each sample was examined individually to remove low-quality cells and cell contaminations. Genes detected in more than three cells were retained, and cells expressing fewer than 200 distinct features were removed. Then, for each sample, data were normalized and scaled and cells were clustered following the standard Seurat protocol. The remaining contaminations were identified using the SingleR package (v1.4.1). The detailed metadata (including patient identifier and CMV status) were retrieved from the original studies. For dataset 4, because the original data were enriched at a ratio of 1:1 between NKG2C^+^ and NKG2C^−^ NK cells, the samples were downsampled to match the initial biological ratio of each sample (donor). For datasets 5, 6 and 7, the preprocessed Seurat objects were used.

### Batch-effect correction and unsupervised clustering

The samples were then merged. To reduce the batch effect during the clustering process, the 11,965 genes present in each of the samples were kept for the clustering step of the analysis. To account for the difference in sequencing depth between samples, count data were normalized using the Multibatchnorm function with the parameter ‘batch= sample’ of batchelor (v1.10.0). The top 5,000 highly variable genes (HVGs) were identified in each sample using the FindVariableFeatures function in Seurat (v4.0.0). Then, to choose the 2,000 best features to keep for integration, the SelectIntegrationFeatures function of Seurat was used with the parameter setting ‘nfeatures = 2000’. Gene expression was then scaled and centered using the ScaleData function of the Seurat library. Next, PCA was performed on the HVG matrix to reduce noise and reveal the main axes of variation using the RunPCA function, and the top 30 components were retained for analysis. The batch effects were corrected using harmony (v0.1.0) correction algorithm across samples^55^. UMAP dimensional reduction and the shared nearest neighbor graph were calculated on harmony-corrected PCA embeddings. The resolution parameter of the FindClusters function of Seurat was chosen to maximize the mean sc3 stability of the clustering for a granularity ranging from *k* = 0.5 to *k* = 1.4. The cluster of proliferating cells was identified using the CellCycleScoring function of Seurat. Cells in these clusters were then removed, and a new UMAP visualization was calculated to better visualize the remaining clusters and cells. The final object used for the analysis of datasets 1–4 is available at: https://collections.cellatlas.io/meta-nk.

The cluster-specific marker genes were identified using the FindAllMarkers function of Seurat with the parameter ‘method= wilcox, only.pos = TRUE, min.pct = 0.2, logfc.threshold = 0.25’.

### Scoring with signatures

To score cells with respect to specific signatures, the top 20 cluster-specific markers (calculated as defined above) were entered into the AddModuleScore function. In brief, the mean expression level for each gene in the defined expression programs was calculated for each cell, and the aggregated expression of control gene sets was then subtracted. All analyzed genes were binned on the basis of the mean expression level, and control genes were randomly selected from each bin.

### RNA-velocity analysis

To limit batch effects and to take into account the differences in the quality of the samples, the RNA-velocity analysis^56^ was carried out separately on the different samples. First, the spliced and unspliced unique molecular identifiers were recounted using the Python package velocyto^57^ (v0.2.2). Subsequently, RNA velocity was estimated using the scvelo function implemented in the R package velociraptor (v3.18). Velocity calculations were restricted to genes previously used for data integration. To facilitate visualization, velocity pseudotimes were projected onto the UMAP coordinates.

### Diffusion-map analysis

Diffusion-map algorithms implemented in the R package destiny^19^ (v3.4.0) were used to infer pseudotime. We removed NK3 cells from the analysis owing to their specific quasi-clonal dynamic. To eliminate the dataset batch effect, the analysis was performed on the biggest dataset (dataset 4) alone. To prevent individual batch effect (at the sample level), the RunFastMNN function implemented in the R package batchelor^58^ (v1.10.0) was used. The corrected expression matrix was then used as input to generate diffusion maps using the DiffusionMap function with the parameters set to ‘censor_val = 30, censor_range = c(30,40)’. The Destiny algorithm automatically identified three ‘root’ cells. We selected the first root cell as the main root because it is located at the start of the directed streamline inferred by RNA velocity, and we then calculated the diffusion pseudotime for all the cells using the DPT function.

### Transcriptional trajectory analysis

To confirm the identified transcriptional trajectories and to better understand the changes along the trajectory from NKint to NK1C, we performed pseudotime analysis using Monocle3 (ref. ^20^) v1.3.1 on every sample from datasets 1–4a together. NK3 and NK2 cells were removed from the analysis, to focus only on the NK1-maturation process. The learn_graph function was run with the parameter ‘ncenter = 150’ to prevent over-branching of the trajectory. The starting point of the trajectory was chosen as the endpoint of the branch in the NKint population, as identified by the RNA-velocity and diffusion-map analyses. The pseudotime was then calculated using the order_cells function. Then, we performed Moran’s *I* test to detect significant genes showing correlation along the principal graph, selected the top 150 genes with a *q* value < 0.05 and the highest Moran’s *I* correlation score and plotted their expression (*z* score) along the pseudotime using the Heatmap function of the ComplexHeatmap library (v2.6.2).

### SCENIC analysis

Activated regulons in the different subsets were analyzed by SCENIC^23^ (v0.12.1). The data analyzed for the identification of the main six NK subpopulations was used as input for the python implementation of the SCENIC algorithm (pyscenic)^59^. In brief, the gene–gene co-expression relationships between transcription factors and their potential targets were inferred using the grn function with the gene regulatory network reconstruction algorithm ‘grnboost2’ selected. A transcription factor and its target genes together make up a regulon. Then, ctx was used to refine the regulons by using targets that do not have an enrichment for a corresponding motif of the transcription factor, effectively separating direct from indirect targets on the basis of the presence of a *cis*-regulatory footprint. Next, the command aucell was used to calculate the regulon activity for each cell. Then, the list of regulons was cross-checked with a robust database of verified transcription factors^24^ to remove unreliable transcription factors and proteins that bind to RNA and DNA non-specifically, and to limit the analysis to bona fide transcription factors. The regulon activity was then scaled and centered before visualization using the Heatmap function of the ComplexHeatmap library.

### ENKP signatures scoring

To score the cells with ENKP-derived NK cell signatures, we extracted the lists of the most representative genes differentially expressed in ENKP-derived NK cells and converted them to their human equivalent. Because they do not have a human equivalent, and owing to their evolutionary convergence, the genes in the Klra family were replaced with the equivalent human KIR genes^60^. Cells were then scored using the AddModuleScore function on the 20 most significant genes. For ILCP scoring, gene signatures were directly retrieved from the original publication^22^.

### GO enrichment analysis

We performed GO enrichment analysis with the clusterProfiler package (v3.18.1). Eight descriptions of interest were chosen among the top 20 most discriminating GO annotations for each cluster. Enrichment scores (*P* values) for the eight selected GO annotations were calculated by a hypergeometric statistical test with a significance threshold of 0.05. The data were plotted as the −log_10_(*P*) values after Benjamini–Hochberg correction. The significance threshold was set at −log_10_(0.05).

### Cytokine responsiveness

To compare cytokine responsiveness across NK cell subsets, we normalized the raw gene counts to log_2_-scaled counts per million, followed by mean centralization to enable direct comparison across cells. The data were then analyzed in CytoSig^14^ (v0.0.3), with the parameter -s 2 to include a more comprehensive set of signatures. *P* values were derived from the comparison of *z* scores between one NK cell subset and the others, using Student’s *t*-tests.

### CITE-seq analysis

For the analysis of CITE-seq data (dataset 5), data that had been preprocessed as described in ref. ^10^ were used. In brief, after removing the cells with an outlier number of features (genes and or ADTs), HTODemux was used to detect and remove doublets^61^. Then, the batch correction was performed using SCTransform followed by the reciprocal PCA workflow^62^. The same process was used for ADTs, but normalization was performed by CLR transformation within each cell. Then, the PCA was run on both RNA and protein modalities, and the top 40 or top 50 dimensions, respectively, were used to construct *k*-nearest neighbor graphs. This graph was then used as input for the WNN procedure^62^. On the basis of the author’s annotations, NK cells were extracted and proliferating NK cells were removed. Cells that arose after vaccination (on days 3 and 7) were also removed from the analysis, so only untreated cells were kept. The remaining 5,708 NK cells were reclustered with very high granularity (*k* = 0.2). Differentially expressed genes and ADTs were identified using the two-sided Wilcoxon rank-sum test with Bonferroni adjustment calculated using the FindAllMarkers function, as described above. For better visualization of ADT expression on the UMAP, the FeaturePlot function of Seurat was used with the parameters ‘min.cutoff = ‘q01’, max.cutoff = ‘q99’’ to prevent outliers from affecting the color scale too strongly.

### Optimization of clustering

To determine the most appropriate granularity of clustering in an unbiased way, the clustree package was used to quantify the SC3 stability metric. This metric is used to evaluate clustering stability at various levels of detail^63,64^. This approach measures how consistently cell groupings hold up across different clustering resolutions and quantifies the stability of each cluster at chosen levels of granularity (Extended Data Fig. 3a). By pinpointing the granularity that maximized SC3 stability, we determined the most reliable clustering configuration (Extended Data Fig. 3b), ultimately settling on a granularity value of 0.7, which corresponded to 11 clusters (Extended Data Fig. 3c).

### Metabolic-pathway analysis

To compare metabolism across subsets, the scGSVA (https://github.com/guokai8/scGSVA), which is the single-cell implementation of GSVA^65^, was used. For this study, only the major metabolic pathways for which multiple genes were sufficiently detected (for example detected in more than 10% of the cells in at least of the NK populations) were retained.

### Enhanced identity prediction through label transfer

To obtain classifications of NK1, NK2 and NK3 cells in dataset 7, we used Seurat’s established protocol for label transfer. Initially, a reference was constructed using datasets 1–4, enabling the annotation transfer to dataset 7. Of note, NKint cells were categorized as NK1, reflecting their initial position in the NK1-maturation trajectory. Through the integration and label transfer process (see https://satijalab.org), we examined the method’s reliability and annotation precision by applying it to datasets 1–4. We then assessed the labeling accuracy on a 20% subset of each population, which was excluded from reference training (Extended Data Fig. 9a). This evaluation demonstrated a minimum prediction accuracy of 86% across populations, with NK1 identification being particularly accurate (90.7% accuracy). The integrity of label transfer to dataset 7 was further assessed by examining the highest prediction score for individual cells within both blood and tumor environments (Extended Data Fig. 9b,c). This confirmed that the NK1 population was the most confidently predicted. Additionally, we assessed the cells’ congruence with NK1, NK2 and NK3 signatures, grouping them by their predicted identities to confirm the enrichment of each predicted population with its corresponding signature (Extended Data Fig. 9d,e).

### PCA and covariance analysis

For PCA analysis on dataset 7, a structured three-step approach was adopted. Initially, cells were categorized by tissue type (tumor or blood) and cancer classification. Following normalization, the top 2,000 variable features within each category were identified using the FindVariableFeatures function. Subsequently, the FindIntegrationFeatures function pinpointed the 2,000 most variable genes across categories. Post-scaling, we computed the mean expression of these 2,000 selected features for cell groups, classified by predicted identity, cancer type and tissue, using Seurat’s AverageExpression function. The data were then scaled again for PCA analysis, which was conducted with the ade4 library. This procedure was replicated for tumor and blood NK cells independently and included a Kruskal–Wallis test to determine the principal components (PC2 and PC3) that best differentiated the three primary NK cell populations in both blood and tumor contexts. For covariance analysis, the same preparatory steps were used, followed by calculation of the Spearman correlation among each group using the cor function. The Pheatmap package was used for the visualization of these correlations.

### Reporting summary

Further information on research design is available in the Nature Portfolio Reporting Summary linked to this article.

## Online content

Any methods, additional references, Nature Portfolio reporting summaries, source data, extended data, supplementary information, acknowledgements, peer review information; details of author contributions and competing interests; and statements of data and code availability are available at 10.1038/s41590-024-01883-0.

### Supplementary information

Supplementary Information

Reporting Summary

Supplementary Table 1 Differentially expressed genes.

Supplementary Table 2 Summary of cluster proportions.

Supplementary Table 3 Dataset presentation.

## Data Availability

All the scRNA-seq and CITE-seq data used in this study have been deposited in the Gene Expression Omnibus. The accession code for each of the datasets used is listed in Supplementary Table 3. Datasets 1–7 correspond to the following accession numbers, respectively: GSE119562, GSE130430, GSE184329, GSE197037, GSE164378, GSE212890 and GSE240441. Single-cell sequencing data were aligned with the GRCh38 human reference genome. To make our data more accessible to the broader research community, we have created an interactive portal (https://collections.cellatlas.io/meta-nk) designed for easy analysis and visualization of our single-cell data.

## Code availability

All the custom code used in this study has been deposited on GitHub (https://github.com/RebuffetLucas/Meta_NK_Project).
