# Supplementary material for: High-dimensional single-cell analysis of human natural killer cell heterogeneity
Source: Nat Immunol. 2024 Jul 2;25(8):1474–88. doi: 10.1038/s41590-024-01883-0 (PMC11291291; doi:10.1038/s41590-024-01883-0)
Supplement: Supplementary file 1 — Supplementary Information [file 41590_2024_1883_MOESM1_ESM.pdf]

nature immunology

Supplementary information https://doi.org/10.1038/s41590-024-01883-0

High-dimensional single-cell analysis of human natural killer cell heterogeneity

In the format provided by the authors and unedited

Supplemental information

Human circulating NK cells comprise 3 main populations

Using the transcriptional signatures presented in Fig. 1b, we performed module score analysis, which confirmed the robustness of the NK cell classification, as evidenced by the clear delineation of three distinct regions on the Uniform Manifold Approximation and Projection (UMAP) plot (Extended Data Fig. 1a,b). Subsequent analyses indicated that the 4 primary $CD56^{dim}$ subsets identified in the blood in Ref[11] aligned closely with the NK1 profile, while the $CD56^{dim}CD16^{high}$_c8_KLRC2 cluster corresponded with the NK3 signature. Similarly, $CD56^{bright}$ NK cell subsets aligned predominantly with the NK2 signature, especially those clusters expressing *IL7R* at high levels (Extended Data Fig. 1c).

We further corroborated the signature's robustness by applying it to two additional scRNAseq samples from dataset 4 (Extended Data Fig. 2a). Unsupervised clustering also resulted in the identification of the same three NK cell populations. Notably, most of the top 20 most distinctive genes for each population overlapped with those identified in the CITE-seq dataset (Extended Data Fig. 2b), indicating conserved transcriptional profiles.

The distribution of the markers previously identified on the UMAP corresponds to the distribution of the NK1, NK2, and NK3 populations (Extended Data Fig. 2c). Some markers, like *SELL* (encoding CD62L) and *GZMK*, were selective to the NK2 subset, while *SPON2* was unique to NK1, and *IL32* was only seen in NK3. In contrast, other markers were common to multiple populations; for example, *FCER1G* was found in NK1 and NK2 but not in NK3. Conversely, *FCGR3A* was present in NK1 and NK3, but absent in NK2, demonstrating a complex and overlapping pattern of marker expression among the NK cell groups.

The three primary NK cell populations split into six subsets

The determination of cluster quantity is often a subject of debate, prompting us to employ an objective methodology for selecting the optimal number of clusters. We initially mitigated batch effects via Harmony batch correction[55], followed by leveraging the SC3 stability metric, which gauges clustering stability at varying levels of granularity[63, 64]. This method assesses the consistency of cell groupings across a spectrum of clustering resolutions, quantifying the stability of each cluster at selected granularities (Extended Data Fig. 3a). By identifying the granularity that maximizes SC3 stability [63, 64], we ascertained the most reliable clustering configuration (Extended Data Fig. 3b), which led us to select a granularity of 0.7, equating to 11 clusters (Extended Data Fig. 3c). Subsequently, we excluded proliferating and cycling cells (clusters 7 and 9) and any cluster comprising less than 3% of the total cells (cluster 10) in order to focus only on major blood NK cell subsets, resulting in a refined group of eight clusters (Extended Data Fig. 3d).

Transcriptional trajectories of NK cell subpopulations

The detailed examination of NK cell maturation trajectory from NKint to NK1C revealed nine distinct gene modules, each sequentially activated as the cells progressed through maturation stages (Extended Data Fig. 6a). The first module (module 1) encompassed key markers of the NKint subset, including *XCL1*, *XCL2*, *GZMK*, and *KLRC1*, alongside the transcription factor *TCF7*. It also featured genes involved in protein synthesis and regulatory processes, such as the *EEF1* gene family, and those regulating gene expression like *GAS5*. Notably, this module also included previously undefined markers for this subset, such as *CD27* and the chemokine receptors *CXCR6*. Module 2 marked an upregulation in major signaling pathways, highlighted by phosphatases *DUSP1* and *DUSP2*, NKFB signaling, and MAP kinase signaling. This module appeared to be influenced by transcription factors like *NR4A2*, *FOS*, and *JUN*. It suggested the

NKint subset's production of amphiregulin *AREG* and the strong expression of CXCR4, indicating a potential recent egress from the bone marrow. The presence of the *SNHG* family in both Modules 1 and 2 hinted at a potential regulatory role of long non-coding RNAs during the early stages of NK cell maturation. The fourth module signified a transition towards the NK1A subset, with increased expression of genes such as *KLRB1* (encoding CD161), *NEAT1*, *CD160*, *IL2RB* (encoding CD122), and *CCL3*. Next, Module 5, characterized by the expression of the *ZEB2* transcription factor, included markers such as *CD38* and *CCL4*, delineating the transition to the NK1B subset. As cells progressed further along the developmental pathway, modules 7 and 8 were marked by an enhancement in cytotoxic capabilities (evidenced by genes like *GZMA*, *GZMB*, *PRF1*) and maturation markers (*NKG7*, *CX3CR1*, *FCGR3A*, *SPON2*, *CD247*, *FCER1G*, *CST7*, *FGFBP2*). These modules also indicated significant cytoskeletal remodeling (*ACT*, *CAP*, *ARP* gene families, *CFL1*, *RAC2*, *RHOC*) that could be involved in trafficking and immune synapse assembly, stress response mechanisms (*HSPA5*, *PDIA6*), and protein degradation processes (various cathepsins and proteasome components). The final stages of this trajectory, represented by the latest genes in Modules 7 and 8, showed an increase in KIR genes (*KIR2DL3*) and integrins (*ITGB2*, *ITGB7*).
