# Supplementary material for: High-dimensional single-cell analysis of human natural killer cell heterogeneity
Source: Nat Immunol. 2024 Jul 2;25(8):1474–88. doi: 10.1038/s41590-024-01883-0 (PMC11291291; doi:10.1038/s41590-024-01883-0)
Supplement: Supplementary file 4 — Summary of cluster proportions. [file 41590_2024_1883_MOESM4_ESM.pdf]

Supplementary Table 2: Summary of cluster proportions.

	**Mean**	**SD**
**NK1**	58.9 %	± 12.2%
NK1A	18.9%	± 4.3%
NK1B	14.2%	± 5.2%
NK1C	25.8%	± 6.1%
**NK2**	5.7%	± 4.1%
**NKint**	11.1%	± 3.8%
**NK3**	24.3%	± 13.9%
