# Supplementary material for: High-dimensional single-cell analysis of human natural killer cell heterogeneity
Source: Nat Immunol. 2024 Jul 2;25(8):1474–88. doi: 10.1038/s41590-024-01883-0 (PMC11291291; doi:10.1038/s41590-024-01883-0)
Supplement: Supplementary file 5 — Dataset presentation. [file 41590_2024_1883_MOESM5_ESM.pdf]

Supplementary Table 3: Dataset presentation.

**Dataset**<br>DOI	**Sorting strategy**	**Protocol / Chemistry**	**Number of samples**	**Number of cells after filtering**	**GSE number**<br>Sample ID / HCMV status
**Dataset 1**<br><br>Crinier 2018, DOI: 10.1016/j.immuni.2018.09.009.	CD3- , CD14- , CD19- , CD45+ , CD56+	3’ V2	1	1,467	**GSE119562**<br>GSM3377678
**Dataset 2**<br><br>Yang 2019, DOI: 10.1038/s41467-019-11947-7	CD3- , CD19- , CD14- , CD7+	3’ V2	2	2,807	**GSE130430**<br>GSM3738542<br>GSM3738543
**Dataset 3**<br><br>Witkowski 2021,DOI: 10.1038/s41586-021-04142-6	CD3- , CD19- , CD14- , CD45+ , CD56+	5’ V2	5	7,569	**GSE184329**<br>GSM5584154<br>GSM5584155<br>GSM5584156_1<br>GSM5584156_2<br>GSM5584156_3
**Dataset 4**<br><br>Rückert 2022, DOI: 10.1038/s41590-022-01327-7	CD3- , CD14- , CD19- , CD7+ , NKG2C+ or NKG2C- (combined 1:1)	Dataset 4a<br>3’ V2	5	24,427	**GSE197037**<br>GSM5907303 HCMV^{neg}<br>GSM5907304 HCMV^{neg}<br>GSM5907300 HCMV^{pos}<br>GSM5907301 HCMV^{pos}<br>GSM5907302 HCMV^{pos}
		Dataset 4b<br>3’ V3	2	14,991	**GSE197037**<br>GSM5907305 **HCMV**^{pos}<br>GSM5907306 **HCMV**^{pos}
**Dataset 5**<br><br>Hao 2021, DOI: 10.1016/j.cell.2021.04.048	Based on ADT and authors annotations: CD3- , CD14- , CD19- , CD45+ , CD56+	3’ and 5’ V3 + 228 ADT	8	5,708	**GSE164378**<br>GSM5008737<br>GSM5008738<br>GSM5008739<br>GSM5008740<br>GSM5008741<br>GSM5008742
**Dataset 6**<br><br>Tang 2023, DOI: 10.1016/j.cell.2023.07.034	“In silico sorting”: CD3E- CD3G- , CD3D- , KLRF1+ or NCAM1+	3’ and 5’ V3 and V2	676	84,343 (blood)<br>34,756 (tumor)	**GSE212890**
**Dataset 7**<br><br>Jaeger, under review	Tissue-dependent sorting	3’ and 5’ V3 and V2	19	48,621	**GSE240441**
